# Lysine acetylome profiling uncovers novel histone deacetylase substrate proteins in *Arabidopsis*

**DOI:** 10.15252/msb.20177819

**Published:** 2017-10-23

**Authors:** Markus Hartl, Magdalena Füßl, Paul J Boersema, Jan‐Oliver Jost, Katharina Kramer, Ahmet Bakirbas, Julia Sindlinger, Magdalena Plöchinger, Dario Leister, Glen Uhrig, Greg BG Moorhead, Jürgen Cox, Michael E Salvucci, Dirk Schwarzer, Matthias Mann, Iris Finkemeier

**Affiliations:** ^1^ Plant Proteomics Max Planck Institute for Plant Breeding Research Cologne Germany; ^2^ Plant Molecular Biology Department Biology I Ludwig‐Maximilians‐University Munich Martinsried Germany; ^3^ Mass Spectrometry Facility Max F. Perutz Laboratories (MFPL) Vienna Biocenter (VBC) University of Vienna Vienna Austria; ^4^ Plant Physiology Institute of Plant Biology and Biotechnology University of Muenster Muenster Germany; ^5^ Proteomics and Signal Transduction Max‐Planck Institute of Biochemistry Martinsried Germany; ^6^ Interfaculty Institute of Biochemistry University of Tübingen Tübingen Germany; ^7^ Department of Biological Sciences University of Calgary Calgary AB Canada; ^8^ US Department of Agriculture, Agricultural Research Service Arid‐Land Agricultural Research Center Maricopa AZ USA; ^9^ Present address: Department of Biology Institute of Biochemistry ETH Zurich Zurich Switzerland; ^10^ Present address: Leibniz‐Forschungsinstitut für Molekulare Pharmakologie im Forschungsverbund Berlin e.V. (FMP) Berlin Germany

**Keywords:** *Arabidopsis*, histone deacetylases, lysine acetylation, photosynthesis, RuBisCO activase, Methods & Resources, Plant Biology, Post-translational Modifications, Proteolysis & Proteomics

## Abstract

Histone deacetylases have central functions in regulating stress defenses and development in plants. However, the knowledge about the deacetylase functions is largely limited to histones, although these enzymes were found in diverse subcellular compartments. In this study, we determined the proteome‐wide signatures of the RPD3/HDA1 class of histone deacetylases in *Arabidopsis*. Relative quantification of the changes in the lysine acetylation levels was determined on a proteome‐wide scale after treatment of *Arabidopsis* leaves with deacetylase inhibitors apicidin and trichostatin A. We identified 91 new acetylated candidate proteins other than histones, which are potential substrates of the RPD3/HDA1‐like histone deacetylases in *Arabidopsis*, of which at least 30 of these proteins function in nucleic acid binding. Furthermore, our analysis revealed that histone deacetylase 14 (HDA14) is the first organellar‐localized RPD3/HDA1 class protein found to reside in the chloroplasts and that the majority of its protein targets have functions in photosynthesis. Finally, the analysis of HDA14 loss‐of‐function mutants revealed that the activation state of RuBisCO is controlled by lysine acetylation of RuBisCO activase under low‐light conditions.

## Introduction

Optimal plant growth and development are dependent on fine‐regulation of the cellular metabolism in response to environmental conditions (Nunes‐Nesi *et al*, 2010). During a day or a season, plants often face rapidly changing environmental conditions such as changes in temperature, light intensity, and water and nutrient availability (Calfapietra *et al*, 2015). Due to their sessile life style, plants cannot escape from environmental perturbations. Instead, plants activate a variety of cellular response mechanisms that allow them to acclimate their metabolism to the environment. Cellular signaling networks are activated within seconds when metabolic homeostasis is perturbed, and these networks regulate the plant's physiology (Dietz, 2015; Mignolet‐Spruyt *et al*, 2016). Such signaling networks regulate gene expression, translation, protein activity, and turnover. Post‐translational modifications (PTMs) of proteins like phosphorylation, ubiquitination, methylation, and acetylation play a pivotal role in all of these regulatory processes (Hartl & Finkemeier, 2012; Johnova *et al*, 2016). Except for phosphorylation, most of the cellular protein targets and the regulating enzymes of these PTMs are largely unexplored in plants (Huber, 2007). Here, we study the regulation of lysine acetylation.

Lysine acetylation is a post‐translational modification (PTM), which was first discovered on histone tails where it is now known to regulate chromatin structure and gene expression (Allfrey *et al*, 1964). The transfer of the acetyl group to lysine neutralizes the positive charge of the amino group, which can affect the biological function of proteins such as enzyme activities, protein–protein, and protein–DNA interactions (Yang & Seto, 2008). Acetyl‐CoA serves as substrate for lysine acetylation in an enzymatic process catalyzed by different types of lysine acetyltransferases (KATs) (Kleff *et al*, 1995; Parthun *et al*, 1996; Yuan & Marmorstein, 2013; Drazic *et al*, 2016). However, lysine acetylation can also occur non‐enzymatically especially at a cellular pH higher than eight (Wagner & Payne, 2013; König *et al*, 2014a); a level that can be reached during active respiration in the mitochondrial matrix, as well as in the chloroplast stroma during photosynthesis (Hosp *et al*, 2016). Non‐enzymatic acetylation is of particular abundance in bacteria, which additionally contain the highly reactive acetyl‐phosphate as metabolite (Weinert *et al*, 2013). In plants, many organellar proteins from mitochondria and chloroplasts were previously identified as lysine‐acetylated (Finkemeier *et al*, 2011; Wu *et al*, 2011; König *et al*, 2014a; Nallamilli *et al*, 2014; Smith‐Hammond *et al*, 2014; Fang *et al*, 2015; He *et al*, 2016; Hosp *et al*, 2016; Xiong *et al*, 2016; Zhang *et al*, 2016; Zhen *et al*, 2016).

Lysine acetylation can be reversed by lysine deacetylases (KDACs), which were named histone deacetylases (HDA/HDAC) before the more recent discovery of non‐histone protein acetylation. KDACs can be grouped into three different families: (i) reduced potassium dependency 3/histone deacetylase 1 (RPD3/HDA1)‐like, (ii) HD‐tuins (HDT), and (iii) silent information regulator 2 (Sir2) (Pandey *et al*, 2002; Alinsug *et al*, 2009; Shen *et al*, 2015). While the RPD3/HDA1 family has primarily been found in eukaryotes, the Sir2‐type deacetylases also occur in bacteria, and the HDT‐type deacetylases only occur in plants. The *Arabidopsis* genome encodes 18 KDACs from the three different families. The largest family comprises the RPD3/HDA1‐like with 12 genes, four genes belong to the HDTs and two to the Sir2 family. The RPD3/HDA1 family can be further subdivided into class I (RPD3‐like), class II (HDA1‐like), and class IV KDACs, of which *Arabidopsis* possesses 6, 5, and 1 putative members, respectively (Pandey et al, 2002; Alinsug *et al*, 2009; Shen *et al*, 2015). Numerous studies have characterized different genes from the *Arabidopsis* KDAC families over the last two decades (Shen *et al*, 2015). In particular, HDA6, HDA9, and HDA19 from class I are the most well studied *Arabidopsis* KDACs and they have been implicated in many important developmental processes such as seed germination, flowering time, as well as plant hormone‐related stress responses (Zhou *et al*, 2005; Benhamed *et al*, 2006; Chen *et al*, 2010; Choi *et al*, 2012; Cigliano *et al*, 2013; Zheng *et al*, 2016; Mengel *et al*, 2017). In terms of protein targets for deacetylation, very little is known about the preferences and targets of the different plant KDACs. While *Arabidopsis* sirtuin 2 deacetylates selected mitochondrial proteins such as the ATP/ADP carrier (König *et al*, 2014b), mainly histone H3 and H4 deacetylation has been studied for the other two families of KDACs (Shen *et al*, 2015).

Here, we report the first comprehensive profiling of putative *Arabidopsis* KDAC targets by using two different inhibitors of the RPD3/HDA1 family. By this approach, we identify several heretofore‐unknown potential targets of the *Arabidopsis* KDACs in the nucleus and other subcellular localizations including plastids. Additionally, by the use of a peptide‐based KDAC‐probe, we were able to identify the first KDAC of the RPD3/HDA1 family, which is active in organelles and regulates the activity and activation state of ribulose‐1,5‐bisphosphate‐carboxylase/oxygenase, the key enzyme in photosynthetic CO_2_ fixation, and the most abundant protein on earth.

## Results

### The *Arabidopsis* leaf lysine acetylome 2.0

**Figure 1:**
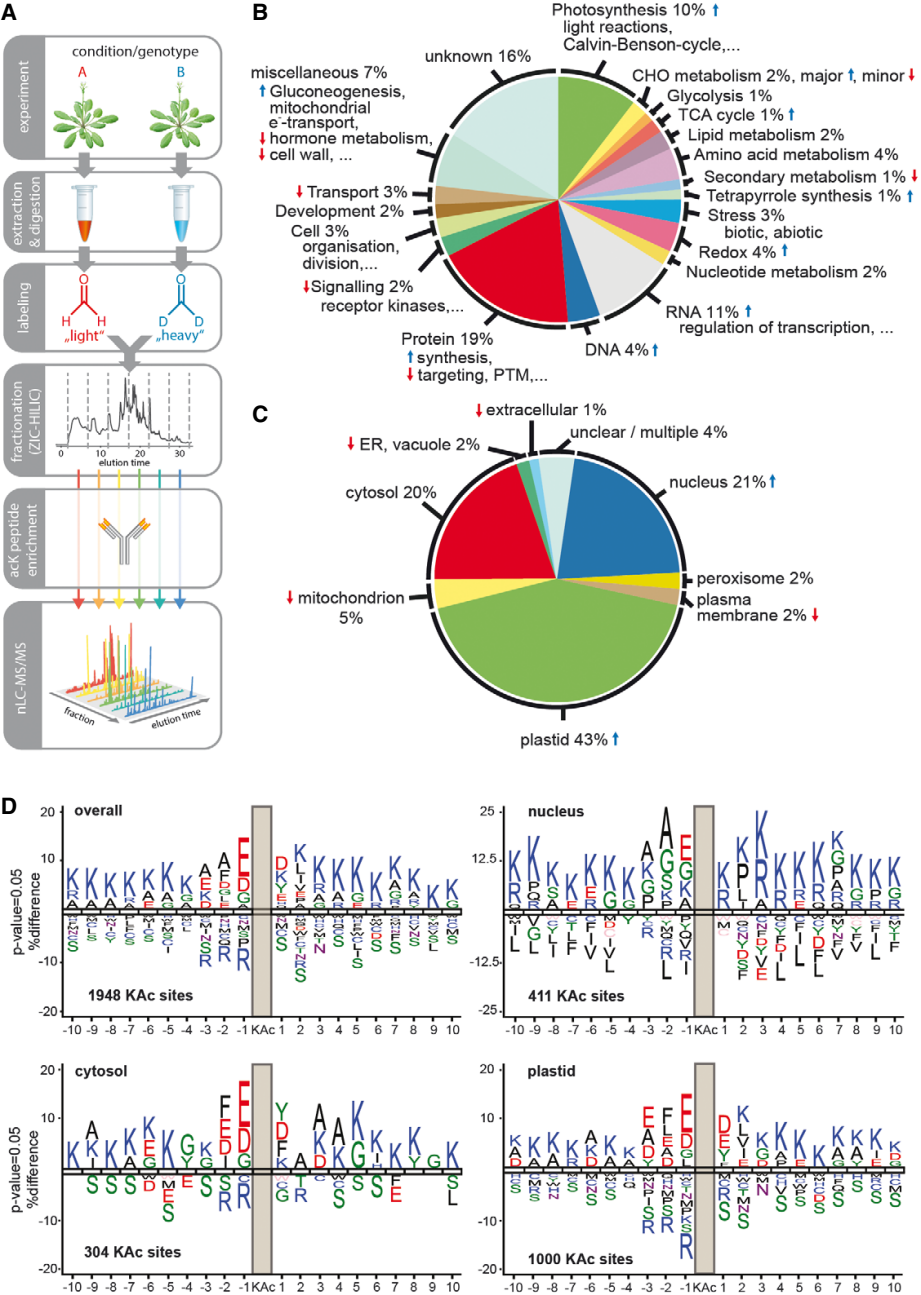
Proteome‐wide identification and classification of the *Arabidopsis thaliana* lysine acetylome AExperimental overview.B, CFunctional classification and subcellular localization of identified lysine‐acetylated proteins. Lysine‐acetylated proteins identified over all experiments were classified according to MapMan categories and SUBA4 localization information, respectively. Over‐ or underrepresentation of categories was determined using a Fisher's exact test with all proteins identified at 1% FDR as background population. Blue and red arrows mark categories significantly enriched at 5% FDR (Benjamini–Hochberg) and a 1.5‐fold‐change cut‐off.DSequence logos for all lysine acetylation sites with all proteins identified as background population (sequence logos were generated using iceLogo, Maddelein *et al*, 2015).

The first two lysine acetylomes of *Arabidopsis* leaves were reported in 2011, with only around 100 lysine acetylation sites identified (Finkemeier *et al*, 2011; Wu *et al*, 2011). Tremendous advances in mass spectrometry, improvements in antibody reagents, and the optimization of the overall protocol now allows a more in‐depth profiling of the *Arabidopsis* lysine acetylome. To be able to quantify acetylome changes upon KDAC inhibitor treatment, we applied an isotopic dimethyl‐labeling approach to differentially label two different protein samples (e.g., treatment and control), combined with an enrichment strategy for lysine‐acetylated peptides (Fig 1A). For this procedure, proteins extracted from leaves were processed and trypsin‐digested via filter‐assisted sample preparation. Peptides were isotopically dimethyl‐labeled, and samples for comparison were pooled. For proteome quantifications, samples were collected at this step and the rest of the sample was further processed by hydrophilic interaction liquid chromatography fractionation to reduce the peptide complexity. Six to seven fractions were collected and used for immuno‐affinity enrichment using anti‐acetyllysine agarose beads. Peptides were further processed for high‐resolution mass spectrometry, and MaxQuant was used for the data analysis.

Altogether the datasets presented here comprise 2,152 lysine acetylation sites (localization probability > 0.75) on 1,022 protein groups (6,672 identified protein groups in total, Table 1, Datasets EV1, EV2, EV3, EV4 and EV5)—this corresponds to 959 novel acetylated proteins and 2,057 novel acetylation sites when compared to the previously published datasets for *Arabidopsis* (Finkemeier *et al*, 2011; Wu *et al*, 2011; König *et al*, 2014a). A MapMan functional annotation analysis (Thimm *et al*, 2004) was used for the classification of the lysine‐acetylated proteins, applying the TAIR mapping and selecting all identified proteins of the proteome analysis as background population. From the different cellular processes, the functional categories photosynthesis, tetrapyrrole synthesis, gluconeogenesis, redox, TCA cycle, as well as DNA and RNA regulation of transcription were identified as overrepresented as determined by a Fisher's exact test, while processes such as hormone metabolism, cell wall, and secondary metabolism were underrepresented (Fig 1B, at 5% FDR and a 1.5‐fold enrichment/depletion cut‐off). Based on the classification of localization of proteins using SUBA consensus (Heazlewood *et al*, 2007), proteins from plastids and nucleus were clearly overrepresented, while proteins from endoplasmic reticulum, vacuole, mitochondrion, plasma membrane, and extracellular space were significantly underrepresented in our dataset (Fig 1C).

**Table 1:** Summary of identified features

		Whole proteome analysis		Acetyllysine‐containing		
Experiment	Description	Protein groups	Peptides	Protein groups	Peptides	Sites
1	Apicidin versus Ctrl	2,384	11,188	538	1,064	1,041
2	TSA versus Ctrl	5,107	32,809	493	1,002	930
3	*hda14* versus WT	2,889	13,755	545	1,133	920
4	*hda14* versus WT low‐light	4,138	27,835	367	756	700
5	*hda14* versus WT thylakoids	2,904	15,064	237	592	546
Total		6,672	47,338	1,022	2,405	2,152

Filters applied: 1% FDR at PSM and protein level, score for modified peptides ≥ 35, delta score for modified peptides ≥ 6, acetyllysine site localization probability ≥ 0.75; contaminants removed.

Additionally, we analyzed the local sequence context around the acetylation sites using iceLogo (Maddelein *et al*, 2015) in combination with the *Arabidopsis* TAIR10 database with all identified proteins as background reference (Fig 1D). Overall, negatively charged amino acids, such as glutamate and aspartate, were significantly enriched in the −1, −2, −3 as well as +1 positions surrounding the lysine acetylation site. In more distant positions, lysine residues were the most strongly enriched on either side of the lysine acetylation site. The sequence motif surrounding the lysine acetylation site appeared different depending on the subcellular localization of the respective proteins. For example, the negatively charged amino acids were more prominent on cytosolic and plastidial proteins in comparison with nuclear proteins, as well as the presence of a phenylalanine at position −2. Tyrosine at position +1 was found on cytosolic and plastid proteins, while phenylalanine at position +1 was only found enriched on cytosolic proteins. Interestingly, on the nuclear sequence motifs only positively charged amino acids were found at position +1 as well as generally more neutral amino acids such as glycine and alanine at positions −1 to −3, which are dominating on histone sequence motifs (Fig 1D).

**Figure 2:**
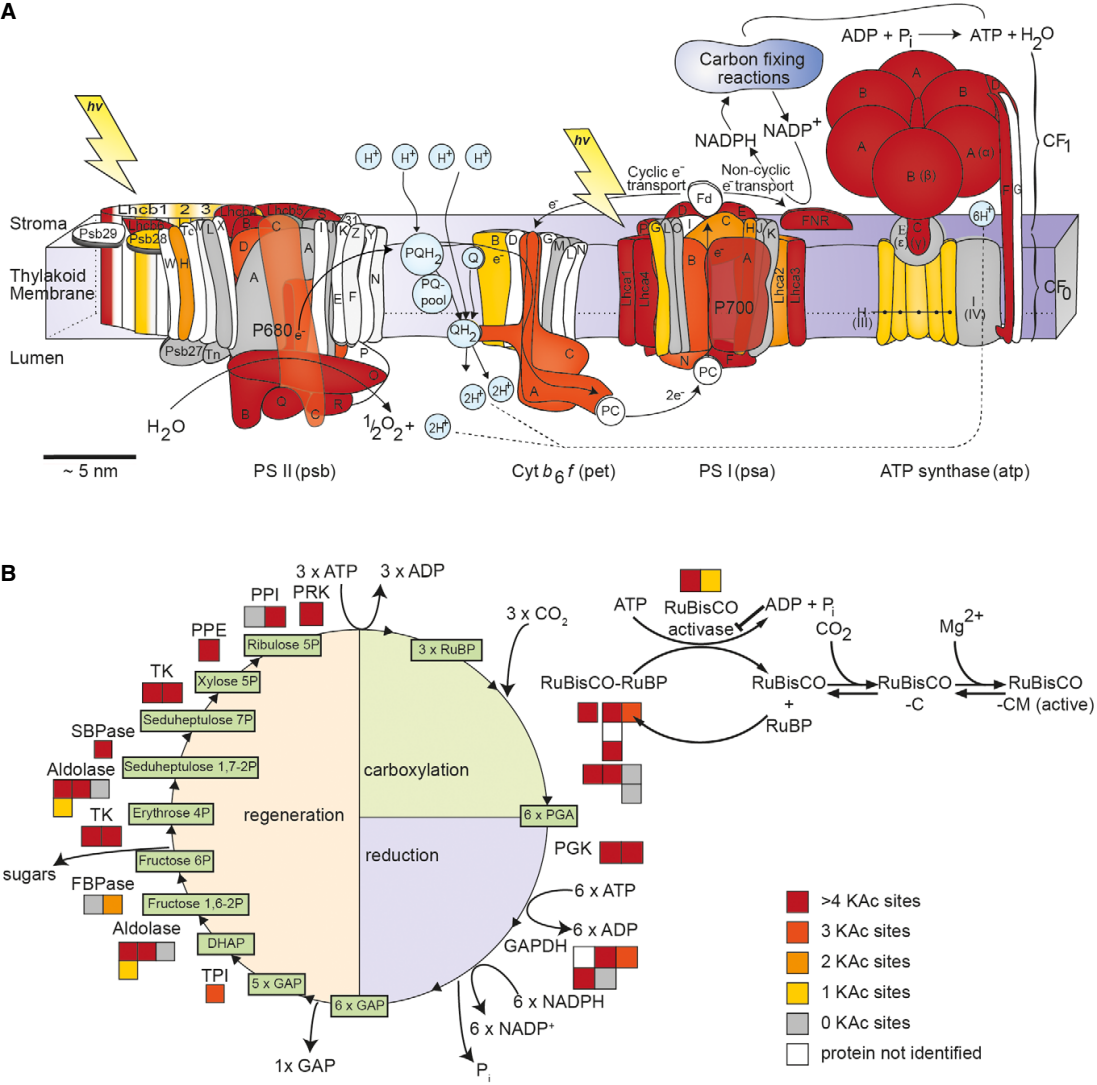
Overview of lysine‐acetylated proteins in the light reactions (A) and the Calvin–Benson cycle (B) identified in this study in *Arabidopsis* A, BThe classification of proteins into functional bins was performed using MapMan (Thimm *et al*, 2004). Color code: proteins not identified in the LC‐MS/MS analyses (white), proteins without identified lysine‐acetylated sites (gray), and proteins with one (yellow), two (orange), three (dark orange), or four or more acetylation sites (red). For the Calvin–Benson cycle, each box indicates a separate *Arabidopsis* AGI identifier as indicated in Dataset EV6. Cytb_6_f, cytochrome b_6_f; FBPase, fructose‐1,6‐bisphosphatase; GAPDH, glyceraldehyde‐3‐phosphate dehydrogenase; PGK, phosphoglycerate kinase; PPE, phosphopentose epimerase; PPI, phosphopentose isomerase; PRK, phosphoribulokinase; PSII, photosystem II; PSI, photosystem I; RuBisCO, ribulose‐1,5‐bisphosphate‐carboxylase/oxygenase; SBPAse, seduheptulose‐1,7‐bisphosphatase; TPI, triose phosphate isomerase; TK, transketolase. A template of the light‐reaction schematic was kindly provided by Jon Nield and modified.

Since 43% of all identified lysine‐acetylated proteins are putative plastid proteins, we further analyzed the distribution of those proteins and number of acetylation sites in photosynthesis (Fig 2). About 24% of the proteins from the photosynthetic light reactions were acetylated on at least four lysine residues (Fig 2A). Proteins from the light harvesting complexes (LHC) of both PSII and PSI were heavily acetylated with 29 lysine acetylation sites on LHCII and 16 on LHCI proteins (Fig 2A). All enzyme complexes involved in the carbon fixing reactions (Calvin–Benson cycle) as well as RuBisCO activase (RCA) contained four or more lysine acetylation sites. With 18 lysine acetylation sites, the large subunit of RuBisCO was the most heavily modified of all the proteins (Fig 2B).

### Identification of novel lysine acetylation sites targeted by *Arabidopsis* RPD3/HDA1‐type KDACs

**Figure 3:**
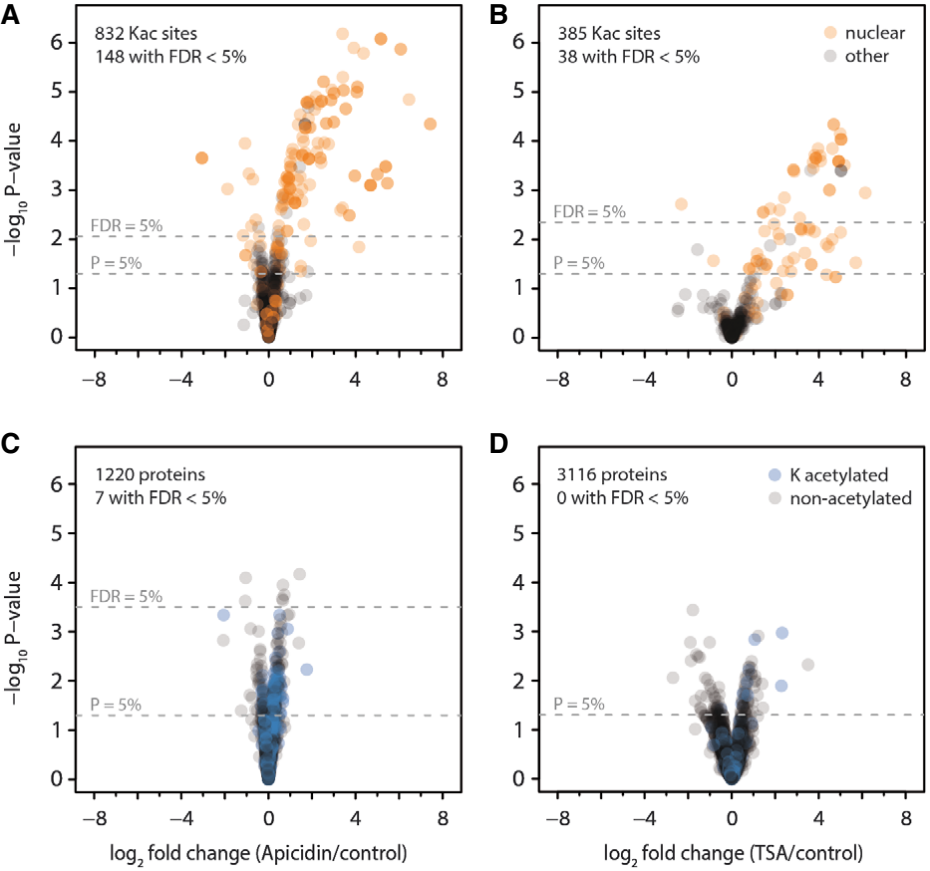
Differential lysine acetylation and protein expression in *Arabidopsis* leaves after inhibitor treatment A–DVacuum infiltration of leaf strips with solutions containing either of the two deacetylase inhibitors apicidin (A, C) or trichostatin A (B, D) versus a buffer control for 4 h leads to differential accumulation of lysine acetylation sites. Volcano plots depict lysine acetylation site ratios (A, B) or protein ratios (C, D) for inhibitor treatment versus control, with *P*‐values determined using the LIMMA package. Orange, protein with nuclear localization according to SUBA4 database. Blue, proteins with lysine acetylation sites identified. Dashed lines indicate significance thresholds of either uncorrected *P*‐values < 5% or Benjamini–Hochberg corrected FDR < 5%. A missing line indicates that the significance threshold was not reached by any of the data points.

Different types of lysine deacetylase inhibitors have been developed in the past decade, which are widely used to modulate the activities of human KDACs in diseases (Newkirk *et al*, 2009). Here, we selected two commonly used KDAC inhibitors, apicidin and trichostatin A (TSA), to target the RPD3/HDA1‐type family of KDACs and to profile their potential protein substrates. While apicidin was shown to specifically inhibit class I KDACs, TSA was described as a general inhibitor of class I and class II KDACs in HeLa cells (Scholz *et al*, 2015). For inhibitor treatment, *Arabidopsis* leaf strips were infiltrated either with a mock control or with 5 μM apicidin and 5 μM trichostatin A (TSA), respectively. Experiments were performed in three independent biological replicates, and the leaf strips were incubated for 4 h in the light before harvest. The protein intensities of the biological replicates had a Pearson correlation coefficient of > 0.87–0.98 (Appendix Fig S1), which indicates the robustness of the approach. Site‐specific acetylation changes were quantified (Fig 3A and B) in addition to changes on total proteome level as control (Fig 3C and D). No significant changes in the regulation of protein abundances were observed after the inhibitor treatments, which covered about 67–88% of proteins carrying the identified acetylated sites (Appendix Fig S2). However, the whole proteome analysis did not cover very low abundant proteins without enrichment. Therefore, we cannot exclude that the other sites, for which we were not able to quantify protein ratios, were not regulated due to bona fide stoichiometry differences from inhibited KDAC activity. However, we restricted inhibitor treatment to 4‐h incubation time in order to minimize potential changes in protein abundances that might result from KDAC‐dependent alterations in gene expression.

For apicidin treatment, 832 lysine acetylation sites were quantified, of which 148 were significantly regulated according to a LIMMA statistical analysis with a FDR cut‐off < 5% (Fig 3A; Dataset EV1). As expected for a KDAC inhibitor treatment, most of the lysine acetylation sites (136 in total) were up‐regulated (log2‐FC 0.4–7.4) after apicidin treatment. The 12 down‐regulated lysine acetylation sites comprise mainly multiply acetylated peptides for which peptide variants of lower acetylation status show a down‐regulation of particular sites, whereas the corresponding peptide with higher acetylation status shows up‐regulation in comparison. Interestingly, while the overall 832 lysine acetylation sites were detected on proteins from various subcellular compartments, 139 of the regulated lysine acetylation sites were found on proteins exclusively localized to the nucleus, such as histones, HATs, proteins involved in the regulation of transcription and signaling (G‐protein and light signaling), DNA‐repair and cell cycle, as revealed from a SUBAcon analysis (Dataset EV1). Three up‐regulated lysine acetylation sites were detected on plastidial proteins including proteins involved in the light reactions (K99, PSAH‐1; PSAH‐2 log2‐FC 0.43) as well as in the Calvin–Benson cycle (K305, SBPase, log2‐FC 0.61). Looking at a less stringent *P*‐value cut‐off < 0.05 (Fig 1B), 182 lysine acetylation sites were found up‐regulated of which 29 were found on organellar proteins. While most of these 29 lysine acetylation sites occur on proteins from the plastids, they only show a rather small increase in acetylation level (log2‐FC 0.2–0.8) (Dataset EV1).

After TSA treatment, only 37 sites of the 385 quantified lysine acetylation sites were significantly up‐regulated with an FDR < 5% (log2‐FC 1.4–6.1) (Fig 3B, Dataset EV2). This low number of regulated sites, compared to apicidin treatment, was mainly due to a higher variability in the biological replicates of the TSA treatment. Of the 37 up‐regulated lysine acetylation sites, only one was detected on a protein with a plastidial localization (K165, At3g17930, log2‐FC 3.6). Analyzing the data with a less stringent *P*‐value cut‐off (*P* < 0.05) resulted in 72 up‐regulated lysine acetylation sites with an average log2‐FC of 0.8–6. Among those were two more plastidial proteins, the RCA β1‐isoform (K438, log2‐FC 1.37) and PSAD‐1/2 (K187/K191, log2‐FC 1.77). Among the common nuclear targets of apicidin and TSA were several histone proteins. In total, 19 regulated lysine acetylation sites were found on histone 2A (H2A) and histone 2B (H2B) proteins. On HTB1 two different lysine acetylation sites were specifically and strongly (log_2_FC at least 2) up‐regulated either upon apicidin (K39, K40) or TSA (K28, K33) inhibition (Appendix Fig S3, Dataset EV1A, Dataset EV2A). The same was true for other histones of the H2B family (HTB9, HTB2), which also showed unique up‐regulated sites depending on the inhibitor used. Interestingly, histones of the H2A family showed the same up‐regulated lysine acetylation sites upon apicidin and TSA treatment. Overall, between apicidin and TSA treatment, there was an overlap of 25 protein groups (*P*‐value < 0.05), which showed enhanced lysine acetylation sites after both treatments (Dataset EV1A, Dataset EV2A). The KDAC inhibitor study revealed that most of the RPD3/HDA1 classes KDACs of *Arabidopsis* have their potential substrate proteins in the nucleus, but that some members also seem to have their targets in other subcellular compartments, such as the plastids.

### HDA14 is the first member of a RPD3/HDA1‐family protein to be localized in organelles

**Figure 4:**
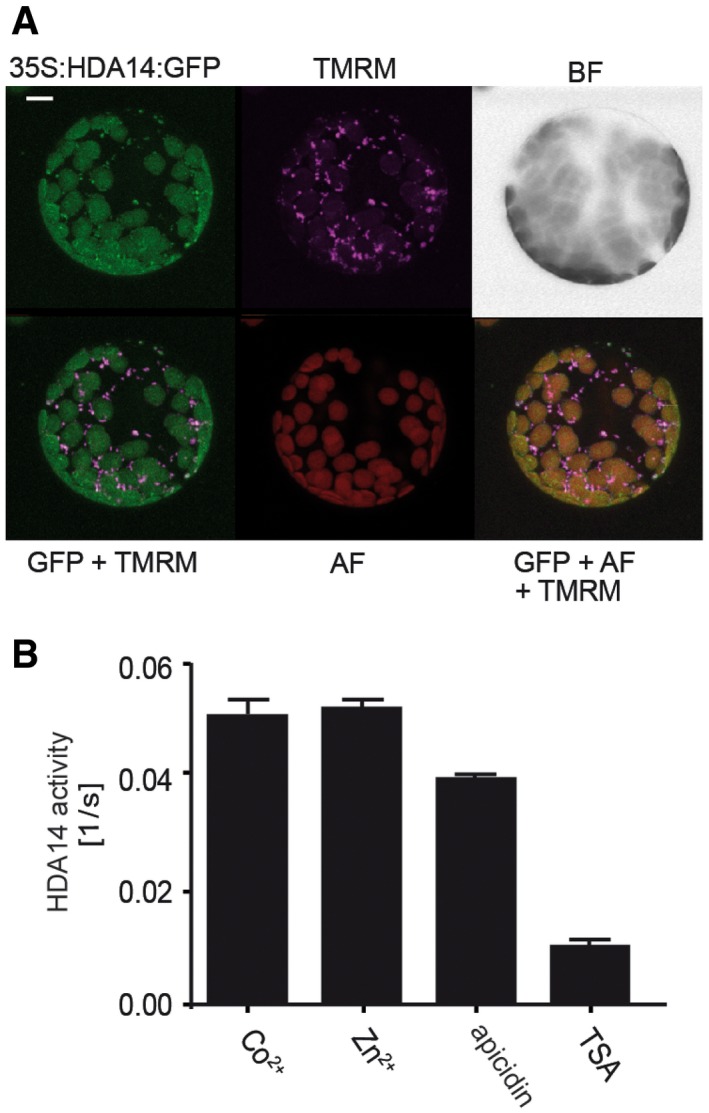
HDA14 protein localizes to the chloroplasts and mitochondria in *Arabidopsis*, and its activity is dependent on cofactors and can be inhibited by deacetylase inhibitors GFP localization (green) of the HDA14‐GFP fusion constructs in *Arabidopsis* protoplasts (35S:HDA14:GFP) from stable transformants. The mitochondrial marker TMRM is depicted in purple. GFP+TMRM shows the overlay image of 35S:HDA14:GFP and TMRM, AF indicates the chlorophyll autofluorescence and BF the bright‐field image of the protoplast. GFP+AF+TMRM represents the overlay image of the three fluorescence channels. Scale bar: 10 μm.Deacetylase activity of the recombinant 6xHis‐HDA14 protein using a colorimetric assay. Co^2+^ and Zn^2+^ were used as cofactors, apicidin (100 μM) and trichostatin A (5 μM) as deacetylase inhibitors (*n* = 5, ± SD).

Members of the RPD3/HDA1 family are usually localized in the nucleus and/or in the cytosol. Here, we had clear indications that proteins targeted to the chloroplast were found hyper‐acetylated upon inhibitor treatment. However, it was not clear whether the hyper‐acetylation already occurred due to KDAC inhibition in the cytosol during transit of the proteins to the plastid or whether there exists a plastid‐localized member of the RPD3/HDA1‐class. Since KDACs are low abundant proteins, they are usually not detected in leaf proteomes and therefore need to be enriched before detection. Here, we used a recently developed peptide‐based KDAC‐probe, mini‐AsuHd (Dose *et al*, 2016), to pull‐down active RPD3/HDA1‐class KDACs from leaf extracts and isolated chloroplasts, respectively, in comparison with the mini‐Lys probe as background control (Table 2). The mini‐AsuHd probe contains a hydroxamate moiety spaced with five carbon atoms to the peptide backbone, which chelates the catalytic Zn^2+^ ion of RPD3/HDA1 family‐HDACs with nanomolar affinities (Dose *et al*, 2016). The mini‐Lys probe contains a lysine residue instead. Three different *Arabidopsis* KDACs were identified in total leaf extracts (HDA5, 14, 15), while in isolated chloroplasts, only HDA14 was identified (Table 2). Although HDA14 contains a predicted target sequence for plastids, it was reported to be localized in the cytosol in a previous study (Tran *et al*, 2012). To confirm the plastid localization of HDA14, we fused GFP to the C‐terminus of the HDA14 protein, instead of the N‐terminus as in the previous study. Protoplasts of the stable transformed 35S:HDA14:GFP plants showed that the signal of the HDA14:GFP fusion protein was overlapping with the autofluorescence of the chlorophyll, as well as with a TMRM signal which visualizes mitochondria (Fig 4A). Hence, these results indicate a dual localization of HDA14 in mitochondria and chloroplasts. To further confirm the results, we performed a Western blot analysis with HDA14 antiserum on proteins from isolated chloroplasts and mitochondria from WT and stably expressing 35S:HDA14:GFP seedlings and detected the endogenous HDA14 as well as the HDA14‐GFP fusion protein in the chloroplast stroma as well as in mitochondria (Appendix Fig S4).

**Table 2:** KDAC pull‐down with mini‐AsuHd probe

Majority protein IDs	Name	Peptides	MS/MS count	Log2‐LFQ CP AsuHd	Log2‐LFQ CP Lys	Log2 enrichment CP	Log2‐LFQ LF AsuHd	Log2‐LFQ LF Lys	Log2 enrichment LF
AT5G61060.1/.2	HDA5	6	11	n.d.	n.d.	n.d.	24.50 ± 0.12	n.d.	> 7^a^
AT4G33470.1	HDA14	7	22	24.74 ± 1.83	19.54 ± 0.05	5.2	26.20 ± 0.2	21.12 ± 0.49	5.1
AT3G18520.1/2	HDA15	2	2	n.d.	n.d.	n.d.	20.84 ± 0.08	n.d.	> 3^a^
ATCG00490.1	RBCL	28	545	33.65 ± 0.36	33.73 ± 0.13	−0.1	33.91 ± 0.09	33.75 ± 0.03	0.2

Selected proteins identified and quantified in pull‐downs by LC‐MS/MS analysis. Protein abundances are expressed as label free quantification (LFQ) values. Numbers indicate mean log2‐transformed LFQ values from two biological replicates of *Arabidopsis* leaves (LF) and isolated chloroplasts (CP). Mini‐Lys probes were used as pull‐down controls to calculate relative enrichments of proteins. LFQ values for RuBisCO are indicated in all samples as background control.

a Estimated enrichment factor assuming a minimum Log2‐LFQ threshold of 17.

### HDA14 is a functional lysine deacetylase and is mainly inhibited by TSA *in vitro*

We produced a recombinant N‐terminally His‐tagged HDA14 protein, which lacks the first 45 amino acids of the predicted N‐terminal signal peptide (Appendix Fig S5), to investigate the predicted KDAC activity of HDA14. The activity of the purified protein was tested in a colorimetric assay based on the deacetylation of a synthetic acetylated p53 peptide coupled to a chromophore (Dose *et al*, 2012). Using this assay, a deacetylase rate of 0.05/s (± 0.0032) was calculated for His‐HDA14 at 100 μM substrate, which is active with both Zn^2+^ or Co^2+^ as cofactors (Fig 4B). Recent publications have shown that recombinant human HDAC8 is more active when the catalytic Zn^2+^ is replaced by Co^2+^ (Gantt *et al*, 2006). However, this is not the case for HDA14, but the enzyme is also active with Co^2+^. Interestingly, apicidin acted only as a weak inhibitor for HDA14 even at concentrations of 100 μM. In contrast, TSA inhibited its activity by 80% at a concentration of 5 μM, the same concentration used in the leaf strip inhibitor experiments.

### HDA14 regulates lysine acetylation levels of plastid proteins related to photosynthesis

**Figure 5:**
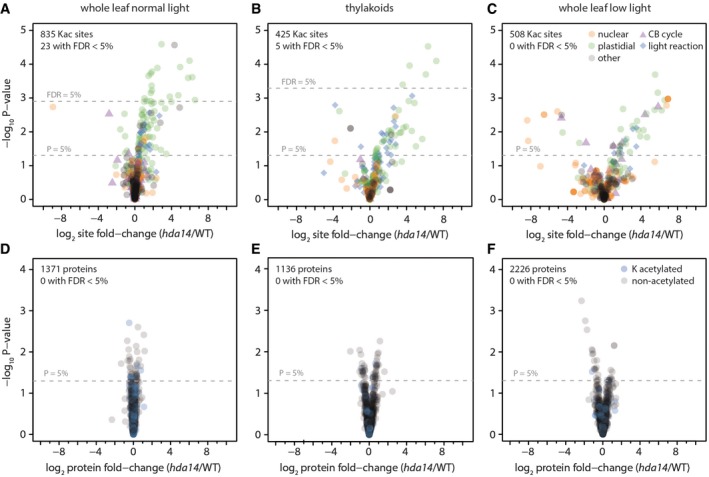
Differential lysine acetylation and protein expression in *hda14* versus wild‐type leaves under normal light (A, D), in isolated thylakoids (B, E), and under low‐light conditions (C, F) A–FVolcano plots depict lysine acetylation site ratios (A–C, top row) or protein ratios (D–F, bottom row) for mutant versus control, with *P*‐values determined using the LIMMA package. Orange, protein with nuclear localization; green, protein with plastidial localization; purple triangles, proteins of the Calvin–Benson (CB) cycle; blue diamonds (top row), proteins of the light reaction; localization information according to SUBA4 database. Blue circles (bottom row), proteins with lysine acetylation sites identified. Dashed lines indicate significance thresholds of either uncorrected *P*‐values < 5% or Benjamini–Hochberg corrected FDR < 5%. A missing FDR line indicates that the 5% threshold was not reached by any of the data points.

To analyze the *in vivo* function of HDA14, a knock‐out line (*hda14*) was obtained (Appendix Fig S6) and changes in lysine acetylation site and protein abundances between *hda14* and WT leaves were compared (Fig 5A–F). In total, 832 lysine acetylation sites were identified and quantified from leaves under normal light conditions (Fig 5A; Dataset EV3), and a further 425 lysine acetylation sites were identified from isolated thylakoids (Fig 5B, Dataset EV4), presumably associated with photosynthetic membrane proteins. While no major changes in protein abundances and plant growth were detected for *hda14* in comparison with WT (Fig 5D–F, Appendix Fig S6), 26 lysine acetylation sites on 26 protein groups were increased in between twofold and 80‐fold in abundance in the mutant with a FDR < 5%. All of these lysine acetylation sites were detected on proteins localized in the plastid. Another 137 lysine acetylation sites from 122 protein groups were found significantly up‐regulated in the mutant but with a lower confidence level (*P* < 0.05). Of these 137 up‐regulated lysine acetylation sites, 35 were uniquely identified in the thylakoid fraction and 13 sites were detected in both pull‐downs. More than 90% of these proteins are annotated as plastid‐localized and are involved in several biochemical processes according to a MapMan analysis (Thimm *et al*, 2004). While around 30% of the proteins have unknown functions, 24% are involved in photosynthesis, 12% in protein synthesis, degradation, and assembly, and around 5% each in lipid metabolism, redox regulation, regulation of transcription, and tetrapyrrole synthesis, as well as 1–3% each are involved in nucleotide metabolism, cell division, ABC transport, secondary metabolism, signaling, organic acid transformation, and amino acid metabolism. Eight of the HDA14 potential target proteins are encoded in the plastome, which further indicates that the deacetylation reaction is occurring within the chloroplast stroma. Among the eight plastome‐encoded proteins affected in their acetylation status by the absence of HDA14, the alpha and beta‐subunit of the ATP‐synthase as well as several photosystem proteins, including the PSII reaction center protein D and the PSI PsaA/PsaB protein, were identified. These results provide a further indication that HDA14 has a regulatory role in photosynthesis.

**Figure 6:**
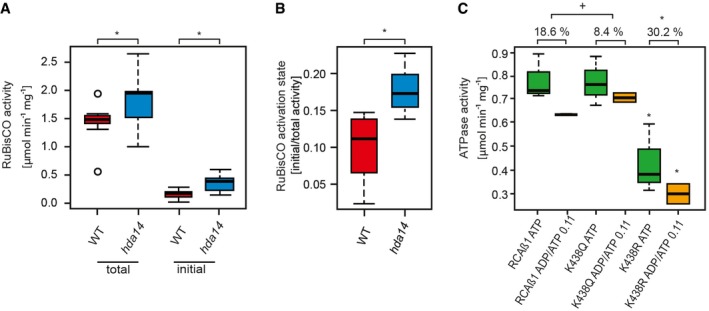
RuBisCO activity and RuBisCO activation state are increased in the *hda14* mutant under low‐light conditions RuBisCO initial and total activity in WT and hda14 in low‐light‐treated plants. Initial activity was measured directly upon extraction. For the total activity, samples were incubated with H_2_CO_3_ for 3 min to fully carbamylate the active site of RuBisCO (*n* = 10, *P < 0.05, *t*‐test).RuBisCO activation state (*P *< 0.05, *t*‐test).ATPase activity of recombinant 6x‐HisRCAβ1 WT, K438Q and K438R with ATP and ADP/ATP = 0.11, respectively (*n* = 3, *P < 0.05, ^+^ *P *< 0.1, *t*‐test). Percentage values on top indicate percent ADP inhibition.Data information: Boxes indicate lower and upper quartiles of data and whiskers indicate highest and lowest values. Small circles represent outliers. The bars across boxes indicate median values.

The regulation of photosynthesis by post‐translational modifications such as phosphorylation and redox regulation is known to be of major importance at low light intensities, for example, during dawn and sunset, when the Calvin–Benson cycle becomes gradually activated or inactivated, respectively, due to changes in stromal pH, ATP, and NADPH levels (Carmo‐Silva & Salvucci, 2013; Buchanan, 2016). Hence, we analyzed the acetylation status of the *hda14* plants in comparison with WT after the plants were transferred from normal light (100 μmol quanta m^2^/s) to low light (20 μmol quanta m^2^/s) intensities for 2 h. Under these conditions, 36 lysine acetylation sites on 32 protein groups showed a significant increase (*P* < 0.05, 2 to 100‐fold), while the total protein abundances of these proteins were unchanged (Fig 5C and F, Dataset EV5). Twenty‐six of these proteins are predicted to be localized in plastids. The MapMan analysis revealed that the biological process photosynthesis is significantly enriched among those regulated proteins, including RCA (K368/K438, log2‐FC 5.89/4.85) as a master regulator of Calvin–Benson cycle activity and RuBisCO large subunit (K474, log2‐FC 1.96) itself. RuBisCO catalyzes the carboxylation or alternatively oxygenation of ribulose‐1,5‐bisphosphate as the first step in either the Calvin–Benson cycle or photorespiration, and thereby enables the photoautotrophic lifestyle of plants. The RuBisCO enzyme is activated by carbamylation of the active site, a process that is dependent on pH, Mg^2+^‐ions and requires the removal of sugar‐phosphate inhibitors that otherwise block the active site (Portis *et al*, 2008). The removal of these inhibitors requires specific conformational changes to RuBisCO that are induced by RCA, a AAA^+^‐ATPase enzyme. RCA is composed of redox‐active alpha isoforms as well as redox‐inactive beta‐isoforms in *Arabidopsis* (Carmo‐Silva & Salvucci, 2013). The RCA activity itself is inhibited under low light by rising ADP concentrations and remains inactive until the photosynthetic electron transport chain again raises the ATP/ADP ratio in response to higher irradiance. The RuBisCO activation state or initial activity, that is, the percentage of active sites free to perform catalysis, as well as total potential activity can be measured by rapid leaf protein extractions (Carmo‐Silva *et al*, 2012). Hence, we determined the RuBisCO activity as well as the RuBisCO activation state of the *hda14* plants compared to WT. The results clearly demonstrate that the RuBisCO initial as well as total activity is significantly increased in the *hda14* mutant compared to WT (Fig 6A). While the total activity was increased on average by around 30%, the initial activity was more than doubled in *hda14* compared to WT (Fig 6A), leading to a significantly 90% increased RuBisCO activation state in the mutant under low light (Fig 6B).

Since the lysine acetylation site K438 of the RCA β1‐isoform was also found increased after TSA treatment (but not K368), we performed a site‐directed mutagenesis on this site in a N‐terminally His‐tagged RCA‐β1 protein. Lysine 438 was exchanged to glutamine (K438Q) and arginine (K438R) to mimic and abolish the lysine acetylation status, respectively. The ATPase activities of the purified mutant RCA proteins were compared to the unmodified WT‐like RCA‐β1 protein (Fig 6C). Strikingly, the total activity of the K438Q mutant was not affected by this mutation, while the replacement of lysine to arginine led to a strongly diminished enzyme activity. Under low‐light conditions, the increase in plastid ADP level plays an important role in the regulation of the RCA activity. Hence, we tested the level of ADP inhibition on the three RCA variants. While the WT‐like isoform was inhibited by nearly 19% at an ATP:ADP ratio of 0.11, the activity of the K438Q mutant was only inhibited by about 8%. The K438R mutant, which mimics the non‐acetylated state, showed an even stronger ADP inhibition of about 30% under these conditions (Fig 6C). Taken together, the results from this experiment further support that lysine acetylation at K438 leads to a higher RCA and thus RuBisCO activity under low light as observed in the *hda14* mutant.

## Discussion

KDACs have important functions in plant development and acclimation of plants to environmental stresses (Shen *et al*, 2015). So far, these enzymes have mainly been studied with respect to their deacetylase function on histones in plants, despite the large number of different types of lysine‐acetylated proteins detected in recent years (Hosp *et al*, 2016). In this work, we studied the proteome‐wide putative targets of the RPD3/HDA1 class of lysine deacetylases in *Arabidopsis* by relative quantification of the changes in the lysine acetylome after inhibitor treatment of *Arabidopsis* leaves with apicidin and TSA. In total, we detected 2,152 lysine acetylation sites in 4‐week‐old *Arabidopsis* leaves when combining all experiments included in this study. The lysine acetylation sites were found on 1,022 protein groups from all different subcellular compartments and compartment‐specific amino acid motifs surrounding the lysine acetylation sites were detected. Similar to human and *Drosophila* sequence motifs, glutamic acid and glycine can be frequently found at position −1 next to the lysine acetylation site also in *Arabidopsis*, while tyrosine, phenylalanine, but not proline, are also enriched in the *Arabidopsis* motifs at position +1, but with a lower frequency (Choudhary *et al*, 2009; Weinert *et al*, 2011). Generally, the acetylated lysines also occur in lysine‐rich regions in *Arabidopsis* similar to those described for human and fly (Weinert *et al*, 2011).

In HeLa cells, apicidin was identified as an inhibitor of mainly the RPD3‐like KDACs, while TSA inhibits enzymes from both RPD3/HDA1 classes (Scholz et al, 2015). Although we cannot exclude that apicidin and TSA have different specificities for *Arabidopsis* KDACs compared to humans, we observed that recombinant *Arabidopsis* HDA14, which is a HDA1‐like KDAC, is not efficiently inhibited by apicidin but by TSA. This supports the notion that similar specificities of both inhibitors exist for the *Arabidopsis* KDACs as well. In *Arabidopsis*, four KDACs belong to the RPD3‐like group, including HDA1/19, HDA6, HDA7, and HDA9 (Hollender & Liu, 2008). Two additional KDAC genes, *HDA10* and *HDA17*, are closely related to *HDA9*, but the predicted proteins lack a catalytic domain and therefore are probably inactive. Lysine acetylation sites on 91 protein groups were significantly (*P* < 0.05) up‐regulated after apicidin treatment and are therefore most likely substrates of at least one of these four KDACs. The dominant nuclear localization among these proteins fits to the observed localizations of the RPD3‐like KDACs in the nucleus. While HDA1/19, HDA6, and HDA9 were detected mainly in the nucleus, the localization of HDA7 has yet to be determined. Although the predicted HDA7 protein contains both a nuclear localization sequence as well as a nuclear export signal, it is unclear to what extent this protein is active due to its low expression level in most tissues.

From the 91 target protein groups identified upon inhibition with apicidin, only 14 are histone‐like proteins. Hence, we identified 77 new candidate protein groups, which are potential substrates of the RPD3‐like KDACs in *Arabidopsis*. This list of potential target proteins with the exact information on their acetylation sites can be regarded as valuable resource for future studies on the KDAC functions in plant stress response and development. Interestingly, a high mobility group box protein with ARID/BRIGHT DNA‐binding domain (At1g76110) was identified as one of the substrate proteins, which was also regulated upon TSA treatment. These types of proteins have been identified as interaction partners of human HDAC1/2 (Joshi *et al*, 2013). Furthermore, a physical interaction was previously detected between *Arabidopsis* HDA6 and the histone H3.3 (At4g40030) (Earley *et al*, 2006). We identified several peptides of histone H3‐like proteins that were up‐regulated by more than 2‐fold at the positions K9, 14, 18, 23, 27, 36, and 37 after apicidin treatment, but less so after treatment with TSA. Several of these lysine acetylation sites on histone H3 are of great importance for chromatin regulation and remodeling [e.g., (Mahrez *et al*, 2016)]. For example, H3K9 acetylation was found to be associated with actively transcribed genes and has a strong impact on various developmental processes in plants (e.g., Ausín *et al*, 2004; Benhamed *et al*, 2006). Differences in the strength of TSA and apicidin inhibition could be explained by differences in the uptake of the inhibitors into the *Arabidopsis* cells, as well as by differences in the Ki values of the different *Arabidopsis* KDACs for these chemicals. Furthermore, our data indicate that TSA might not be effectively taken up into plastids, since the recombinant HDA14 protein was strongly inhibited by TSA, but only few plastid proteins were affected by TSA treatment of *Arabidopsis* leaves.

In addition to the many new KDAC inhibitor target proteins in the nucleus, there were also several interesting candidate proteins identified in the cytosol, such as the FRIENDLY protein (At3g52140), which is required for correct distribution of mitochondria within the cell. In a previous study, we already demonstrated that two lysine sites, which can be acetylated, regulate FRIENDLY function (El Zawily *et al*, 2014).

After TSA treatment, lysine acetylation sites from unique protein groups were regulated, which were not affected by apicidin treatment, indicating that those sites are specifically regulated by HDA1‐type HDACs. These proteins included RCA‐β1, photosystem I subunit D‐2, ribosomal L6 family protein, S‐adenosyl‐L‐methionine‐dependent methyltransferases superfamily protein, the telomere repeat binding factor 1, and a histone H2B protein (Dataset EV2). Candidates of KDAC proteins from the HDA1‐type group that might be responsible for the regulation of the lysine acetylation sites of these proteins include HDA5, 8, 14, 15, and 18, which cluster together with the human class 2 KDACs (Alinsug *et al*, 2009). By using a hydroxamate‐based KDAC‐probe, which allows the enrichment of active RPD3/HDA1‐class KDACs from protein extracts, we were able to detect HDA5, 14, and 15 in total leaf extracts of 4‐week‐old *Arabidopsis* leaves. By using the same probe, we previously enriched all class 1 and class 2b KDACs from HeLa cells, indicating that the probe is able to bind all types of RPD3/HDA1‐class KDACs (Dose *et al*, 2016). Hence, we conclude that HDA5, 14, and 15 are the most abundant KDACs in *Arabidopsis* leaves. Strikingly, both TSA and apicidin treatment resulted in an increased acetylation of plastid proteins involved in photosynthesis. Here, we identified HDA14 as the first organellar‐localized RPD3/HDA1 class protein which is active as a KDAC and which has the majority of its candidate target proteins in the plastid stroma. At the concentration of apicidin used in our study, HDA14 was not significantly inhibited in its activity, which further supports the observation that apicidin mainly inhibits RPD3‐like KDACs. Hence, the plastid target proteins, which showed mildly increased lysine acetylation after apicidin treatment, might be regulated by an unknown RPD3‐like deacetylase. For example, we identified six lysine acetylation sites on the TROL protein, which is required for anchoring the ferredoxin‐NADP reductase (FNR) to the thylakoid membranes and to sustain efficient linear electron flow in the light reactions of photosynthesis. While K328 and K348 of TROL were more than twofold increased in their acetylation level in the *hda14* loss‐of‐function mutant, only K337 showed a 1.2‐fold increased acetylation after apicidin treatment. Lysine acetylation sites on FNR itself were not significantly regulated upon KDAC inhibition. Here, we identified four lysine acetylation sites on both FNR1 (K287, 290, 321, 325) and FNR2 (K90, 243, 299, 330) isoforms, of which only two have been previously reported (Lehtimaki *et al*, 2014).

By analyzing the acetylome of *hda14* mutants, we were able to identify the unique substrate proteins of HDA14. HDA14 was previously identified as a nuclear/cytosolic protein based on enrichment in the microtubule fraction of a red fluorescent‐tagged version of the protein (Tran *et al*, 2012). However, the N‐terminal location of this tag would hinder the protein from entering the chloroplast. Moreover, the authentic N‐terminus of HDA14 contains a clear signal sequence for the plastids as predicted by bioinformatical analysis (Alinsug *et al*, 2009). By using the Asu‐Hd probe on isolated plastid fractions as well as by using C‐terminal GFP‐tagged fusion proteins, we were able to confirm the predicted plastid localization of HDA14. Furthermore, most of the candidate HDA14 substrate proteins identified reside in the plastids and are involved in metabolism and photosynthesis.

With the analysis of the HDA14‐dependent acetylome, we found that the RCA‐β1 site K438 is a substrate site of HDA14. Increased acetylation of this site reduced the ADP sensitivity of the RCA protein, which plays an important role for the Calvin–Benson cycle activation under low light intensities when the ADP/ATP ratio in plastids is still high (Carmo‐Silva & Salvucci, 2013). Since the alpha‐isoform of RCA in *Arabidopsis* is considerably more sensitive to inhibition by ADP than the beta‐isoform (Carmo‐Silva & Salvucci, 2013), the effect of acetylation of K438 of RCA‐β1 in relieving ADP inhibition might be mediated by RCA‐α through an effect of acetylation on subunit (i.e., alpha–beta) interaction.

Lysine acetylation on *Arabidopsis* RCA was detected in a previous study (Finkemeier *et al*, 2011), but on a different lysine residue and the functional consequences were not studied so far. In addition to increased acetylation, we also observed increased RuBisCO activity under low‐light conditions in the *hda14* mutant, which co‐occurred with increased acetylation at K474 on the RuBisCO large subunit next to the strongly increased acetylation of K368 and K438 on RCA and on carbonic anhydrase (K269, At3g01500). Acetylation on all of these proteins might play an important role in fine‐tuning of RuBisCO activity. In contrast to our results obtained in the *hda14* mutant, two independent previous studies revealed that a decrease in RuBisCO acetylation resulted in a higher activity of the enzyme (Finkemeier *et al*, 2011; Gao *et al*, 2016). However, K474 on the RuBisCO large subunit (RBCL) was not detected in either of these studies, and hence, this site could have a different role than acetylation on any of the other 18 acetylation sites detected here and elsewhere.

In conclusion, in this study, we were able to define the heretofore‐unknown acetylation candidate target proteins of RPD3/HDA1 class HDACs in *Arabidopsis* and specifically those of HDA14, as the first identified RPD3/HDA1 KDACs in organelles. Furthermore, our study revealed that about 10% of the detected lysine acetylation sites can be regulated by these types of KDACs in *Arabidopsis* leaves. Many sites might be specifically regulated under certain environmental or developmental conditions due to changes in KDAC activities, as we observed for low‐light conditions for example. The activity of KDAC themselves might be regulated via post‐translational modifications (Mengel *et al*, 2017) or by change in interaction partners that lead to the formation of different KDAC complexes (Dose *et al*, 2016). Future studies, with more detailed analyses of individual lysine sites in proteins and the analysis of further KDAC mutants and environmental conditions, will allow unraveling this complex network of fine‐tuning of protein functions and interactions by lysine acetylation. Since lysine acetylation sites can act as molecular switches, they could be engineered in plant proteins to regulate cell‐signaling cascades, the expression of certain genes, or to modulate the activities of metabolic enzymes. Furthermore, due to recent advances in advances in CRISPR/CAS technologies, lysine acetylation sites can be used for site‐directed mutagenesis also in crop plants. Modifying these lysine residues to constitute acetylated or non‐acetylated mimics ideally will allow a switching of metabolic activities and outputs that have the potential to enhance plant yields or direct metabolism in a way to enhance the accumulation of metabolic intermediates to increase the nutritional values of crops and thereby indirectly promote human health.

## Materials and Methods

### Plant material and growth conditions

Arabidopsis *thaliana* (Col‐0) plants were grown for 4 weeks in a climate chamber using a 12‐h light/12‐h dark (21°C) photoperiod with a light intensity of 100 μmol quanta m^2^/s and 50% relative humidity. For low‐light treatments, plants were transferred to 20 μmol quanta m^2^/s for 2 h before harvest. For growth on plates, *Arabidopsis* seeds were sterilized and transferred to half‐strength Murashige–Skoog medium supplemented with 0.8% phytoagar. The *hda14* line (SALK_144995C) was obtained from the Nottingham *Arabidopsis* stock center (NASC) and PCR screened according to Salk Institute Genomic Analysis Laboratory instructions (O'Malley *et al*, 2007) using the following primers: HDA14_LP 5′‐GAAACATGTCACGCAAAAATG‐3′, HDA14_RP 5′‐TTTTGTTGGTTTGCTTCTTCG‐3′, and the TDNA primer SALK‐Lb1.3 5′‐ATTTTGCCGATTTCGGAAC‐3′. PCR products were run on 1% agarose Tris–acetate (TAE: 40 mM Tris, 20 mM acetate, 1 mM EDTA, pH 8.0) and visualized by UV illumination upon ethidium bromide staining.

### Trichostatin A and apicidin treatment

About 20 fully expanded leaves from 4‐week‐old *Arabidopsis* plants were pooled and cut into 2‐mm‐diameter leaf slices (for each biological replicate). After vacuum infiltration in effector solutions (three times for 5 min), the leaf slices were incubated at 100 μmol quanta m^2^/s for 4 h. All solutions used for infiltrations were made in 1 mM MES pH 5.5 (KOH). All chemicals were purchased from Sigma‐Aldrich (Gillingham, Dorset, UK). All stock solutions were dissolved in DMSO. Control experiments were then performed with DMSO added in same concentrations without effectors. Leaf material was briefly dried on tissues for harvest and flash‐frozen in liquid nitrogen.

### GFP fusion and plant transformation

Entry clones for Gateway cloning were generated with the pENTR/SD/TOPO vector (Invitrogen™). The open reading frame of *HDA14* (At4g33470) without stop codon from *Arabidopsis* (Col‐0) was amplified from cDNA using the following primers: 5′‐CACCATGTCCATGGCGCTAATTGT‐3′ and 5′‐TAAGCAATGAATGCTTTTGGCTCTC‐3′. LR reactions were performed for recombination into the pK7GW2 vector (Karimi *et al*, 2007). The vector construct was verified by sequencing and transformed into *Agrobacterium tumefaciens* strain C58 followed by floral dip transformation of *Arabidopsis* (Col‐0) plants (Clough & Bent, 1998). Transformants were selected by germination of seeds on MS‐agar plates containing kanamycin (50 μg/ml). Resistant plants were transferred to soil and propagated.

### RNA isolation and RT–PCR

Total RNA of *Arabidopsis* leaves was extracted using Trizol^®^ (Invitrogen™) followed by chloroform extraction, and precipitation with isopropanol and subsequently LiCl_2_. The quality and quantity of the RNA were confirmed on agarose gels and a UV‐spectrometer. Complementary DNA (cDNA) was synthesized from DNase‐treated RNA with SuperScriptIII reverse transcriptase (Invitrogen™) following the manufacturer's instruction and using dT_20._ Real‐time qPCR was carried out in triplicate in an iQ™5 Multicolor Real‐Time PCR Detection System (Bio‐Rad) using iQ™SYBR Green Super Mix (Bio‐Rad) and gene‐specific primers: HDA14‐F 5′‐ATCTGTGGCAGACTCGTTTCG‐3′, HDA14‐R 5′‐TCGCACCTTTCTCATTGGTTC‐3′. Levels of selected transcripts in each sample were calculated using a standard curve method (Finkemeier *et al*, 2013). Expression levels of the HDA14 transcript were normalized to *ACTIN2* (At3g18780) transcript as housekeeping gene using the following primers: actin2‐F 5′‐CTGTACGGTAACATTGTGCTCAG‐3′ and actin2‐R 5′‐CCGATCCAGACACTGTACTTCC‐3′.

### Protoplast isolation and confocal laser scanning microscopy

Protoplast isolation was performed from 4‐week‐old *Arabidopsis* leaves after the tape‐sandwich method (Wu *et al*, 2009). Staining with 20 nM TMRM (Sigma) was performed according to the manufacturer's protocol. Imaging was performed with a spectral TCS SP5 MP confocal laser scanning microscope (Leica Microsystems, Mannheim, Germany) using an argon and DPSS‐Laser laser, respectively, at an excitation wavelength of 488 nm (eGFP) and 543 nm (TMRM). The water immersion objective lens HCX PL APO 20.0× 0.70 IMM UV was used for imaging in multitrack mode with line switching. eGFP fluorescence and TMRM fluorescence were measured at 500–530 and 565–615 nm, respectively.

### Heterologous expression and purification of recombinant HDA14 protein

cDNAs were amplified by PCR excluding the coding region for the 45aa signal peptide using the following primers: HDA14p‐F: 5′‐TTTAGTACAGAGAAGAATCCTCTATTACCATCT‐3′ and 5′‐TCAAACAAATTCACCTTATAAGCAATG‐3′. The PCR product was cloned into pEXP‐5‐NT/TOPO^®^ TA (Invitrogen™), which allows expression and purification of the recombinant, N‐terminally 6× His‐tagged protein. Vector constructs were verified by sequencing. After transforming *E. coli* BL21(DE3) (Invitrogen™) with the expression vector, the recombinant protein was expressed using the EnPresso system (BioSilta, Germany) as described before (Jost *et al*, 2015): 500 ml of EnPresso medium was mixed with 12.5 μl ZnCl_2_ (1 mM) solution and 25 μl of the “EnZ I'm” mix. Freshly prepared medium was inoculated 1:100 with a 6‐h‐cultivated pre‐culture at 37°C under gentle shaking (160 rpm) and incubated overnight. Subsequently, the temperature was reduced to 25°C and a “booster tablet” and 50 μl “EnZ I'm” mix were added to the culture medium followed by 500 μM IPTG. The culture was incubated for 24 h at 250 rpm. The cells were harvested by centrifugation (10 min at 3,000 × *g*), and the pellet was resuspended in PBS buffer (pH 8.0) and lysed with a homogenizer (EmulsiFlex‐C5, Avestin) at 4°C. The cleared lysate (centrifugation for 20 min at 30,000 × *g*) was incubated with 1 ml of Ni‐NTA Agarose slurry (Qiagen) for 2 h at 4°C. The resin was washed with 50 ml PBS, pH 8.0, 4°C, and the protein was eluted with 300 mM imidazole in HDAC buffer (8 mM KCl, 100 mM NaCl, 10 mM HEPES, pH 8.0). Pure fractions were combined and dialyzed against HDAC buffer containing 10 mM EDTA and subsequently against HDAC buffer supplied with 0.5 mM EDTA. The sample was concentrated with a centrifugation filter device with 10 kD MWCO (Amicon Ultra, Merck Millipore), supplied with 20% (v/v) glycerol and stored at −80°C until usage.

### HDA14 activity assay

The deacetylation assays were performed with a previously described p53‐derived peptide substrate containing the chromophore 5‐amino‐2‐nitrobenzoic acid (p53‐5,2‐ANB) (Dose *et al*, 2012). To produce the apoenzyme, the purified HDA14 protein was first dialyzed against 10 mM EDTA and 1 mM DTT and in a second step against 0.5 mM EDTA to remove bound metal ions. For the enzyme asssay, HDA14 was supplied with either Zn^2+^ or Co^2+^ ions by incubating the enzyme solution with 1 mM of ZnCl_2_ or CoCl_2_ on ice for 30 min. Deacetylation assays were performed by incubating 1 μM of either Zn^2+^ or Co^2+^ supplied HDA14 with 100 μM p53‐5,2‐ANB substrate in HDAC reaction buffer (10 mM HEPES, 100 mM NaCl, 8 mM KCl, 10 μM BSA, pH 8.0) in a total volume of 50 μl at RT. The reaction was stopped after 10 min by adding 10 μl quenching solution (6.25 μM TSA in 0.1% (v/v) TFA) and developed by adding 10 μl of trypsin solution (6 mg/ml). After 30 min of trypsinization, the reaction mixture was supplied with 70 μl of HDAC reaction buffer, transferred into a 100‐μl quartz cuvette, and the absorbance was monitored at 405 nm in a photometer (Helma, Germany). Studies with inhibitors apicidin (5 and 100 μM) and TSA (5 μM) were performed by adding these compounds to the assay before the reactions were started. All rates were normalized to the concentration of HDA14.

### Western blot analyses

Proteins were separated on 12% SDS–polyacrylamide gels, blotted onto nitrocellulose membrane, and incubated overnight with the primary antibodies. The secondary IRDye 800CW antibody (LI‐COR) was used in a 1:10,000 dilution and detected with the Odyssey reader (LI‐COR).

### RuBisCO activity measurements for activation state determination

For determination of the RubisCO activation state under low‐light conditions, plants were transferred to low irradiation (20 μmol quanta m^2^/s) for 5 h at 21°C, then harvested, and frozen in liquid nitrogen. RuBisCO initial and total activity were assayed by incorporation of ^14^CO_2_ into acid‐stable products (Salvucci, 1992). The leaves were homogenized in extraction medium [100 mM Tricine–NaOH pH 8.0, 1 mM EDTA, 5% polyvinylpyrrolidone (PVP‐40), 5% polyethylene glycol 3350 (PEG3350), 5 mM (DTT), and protease inhibitor cocktail (Roche)]. Initial activities were measured immediately upon extraction, whereas total activities were measured after 3‐min incubation in assays without RuBP to fully carbamylate the enzyme (Carmo‐Silva *et al*, 2012). For each sample, assays were conducted in duplicate. Initial and total activities were used to calculate RuBisCO activation state, that is, (initial/total activity × 100) = % activation.

### Purification and assay of RCA

The coding sequence of the RCAβ1 spliceform (At2g39730.2) was amplified from *Arabidopsis* cDNA using the following primers: 5′‐CTCCGATATCTTACTTGCTGGGCTCCTTT‐3′ and 5′‐TTTTTGATATCTCAAACCTCTGTTTTACC‐3′ introducing SacI and EcoRV restriction sites for cloning into pCDFDuet‐1 (Novagen^®^). Site‐directed mutants of RCA K438R and K438Q were introduced with the QuikChange Site‐Directed Mutagenesis Kit (Agilent Technologies) using the following primers: RCAβ2‐K438R 5′‐GAACTTTCTACGGTAGAACAGAGGAAAAGG‐3′ and RCAβ2‐K438Q 5′‐GAACTTTCTACGGTCAAACAGAGGAAAAGG‐3′. The N‐terminally 6‐His‐tagged protein was expressed and purified from Rosetta‐gami cells (Novagen^®^) as described detail in (Barta *et al*, 2011). ATPase activity of 5 μg recombinant RCA was measured for 1 min at 23°C in 50 μl reaction buffer (100 mM HEPES‐KOH (pH 8.0), 20 mM MgCl_2_) containing 500 μM ATP and 500 μM ATP and 55 μM ADP, respectively. The reaction was heat inactivated at 95°C. The ATP consumption was determined using the KinaseGlo Max Luminescent Assay Kit (Promega) according to the manufacturer's protocol.

### Isolation of intact chloroplasts and mitochondria

Chloroplasts were isolated from dark incubated (12 h) 5‐week‐old rosette leaves of *Arabidopsis*. Leaves were homogenized in ice‐cold HB‐buffer (0.45 M sorbitol, 20 mM Tricine‐KOH pH 8.45, 10 mM EDTA, 10 mM NaHCO_3_, 0.1% BSA, and 2 mM sodium ascorbate). Chloroplasts were purified on a Percoll gradient (40–80%) and resuspended in sorbitol buffer (0.3 M sorbitol, 20 mM Tricine‐KOH pH 8.45, 2.5 mM EDTA, and 5 mM MgCl_2_, 2 mM sodium ascorbate). Mitochondria were isolated as described previously (König *et al*, 2014a).

### Isolation of thylakoids

Chloroplasts were lysed in 2 ml TMK buffer (50 mM HEPES/KOH pH 7.5, 0.1 M sorbitol, 5 mM MgCl_2_, 10 mM NaF), and thylakoid membranes were sedimented at 14,000 × *g*.

### Preparation of cell extracts and enrichment of active histone deacetylases

Leaves from 5‐week‐old *Arabidopsis* plants were homogenized in extraction buffer (50 mM Tris–KOH (pH 7.5), 150 mM NaCl, 10% [v/v] glycerol, 5 mM dithiothreitol (DTT), 1% [v/v] Triton X‐100, and protease inhibitor cocktail (Sigma‐Aldrich). Homogenates were centrifuged at 14,000 × *g*, and protein concentration of the supernatant was determined with the Pierce 660 nm Protein Assay (Thermo Fisher Scientific). All protein extracts were desalted on PD‐10 Desalting Columns (GE Life Sciences), and the samples were eluted with immunoprecipitation buffer (50 mM Tris, pH 7.5, 150 mM NaCl, 10% [v/v] glycerol).

The immobilized peptide probes, mini‐AsuHd, and mini‐Lys (Dose *et al*, 2016) were equilibrated with immunoprecipitation buffer two times and incubated with the protein extracts overnight at 4°C, under constant rotation. The next day, the beads were gently pelleted by centrifugation. The beads were transferred onto micro‐centrifugal filter system (Amchro GmbH) and washed five times with 1 ml immunoprecipitation buffer. Proteins bound on beads were subjected to on‐bead digestion. Proteins were denatured in 6 M urea prepared in 0.1 M Tris–HCl (pH 8.0), 1 mM CaCl_2_ and reduced with 5 mM DTT. Reduced cysteines were alkylated with 14 mM chloroacetamide for 30 min. Excess chloroacetamide was quenched with DTT. Proteins were trypsinated at a urea concentration of 1 M and a trypsin (Sigma‐Aldrich) to protein ratio of 1:100 at 37°C. Resulting peptides were desalted on SDB‐RPS and C18 StageTips, respectively (Rappsilber *et al*, 2007; Kulak *et al*, 2014).

### Protein extraction, peptide dimethyl labeling, and lysine‐acetylated peptide enrichment

Frozen leaf material was ground to fine powder in liquid nitrogen and extracted using a modified filter‐assisted sample preparation (FASP) protocol with 30k MWCO Amicon filters (Merck Millipore) as described in detail in Lassowskat *et al* (2015). Digested peptides were dimethyl‐labeled on C18 Sep‐Pak plus short columns (Waters) as described previously (Boersema *et al*, 2009; Lassowskat *et al*, 2017). Equal amounts of light and medium‐labeled peptides (3–5 mg) were pooled for each replicate and the solvent evaporated in a vacuum centrifuge. The dried peptides were dissolved in 1 ml TBS buffer (50 mM Tris–HCl, 150 mM NaCl, pH 7.6), and pH was checked and adjusted where required. 15 μg peptide mixture was stored for whole proteome analysis. About 10 mg of the pooled labeled peptides was resuspended in 2 ml 95% solvent A (95% acetonitrile, 5 mM ammonium acetate) and 5% buffer B (5 mM ammonium acetate) and fractionated with a flow rate of 500 μl/min on a Sequant ZIC^®^‐HILIC column (3.5 μm, Merck) using a segmented linear gradient of 0–60%. The fractions were combined to seven final fractions and dried in a vacuum centrifuge. Peptides were resuspended in IP buffer (50 mM Tris–HCl pH 7.6, 150 mM NaCl), and the concentration was determined on the spectrophotometer at 280 nm. Lysine‐acetylated peptide enrichment was performed as previously described with 1 mg peptide per fraction (Hartl *et al*, 2015; Lassowskat *et al*, 2017). After enrichment, the eluted peptides were desalted using C18 StageTips and dried in a vacuum centrifuge.

### LC‐MS/MS

Dried peptides were redissolved in 2% ACN, 0.1% TFA for analysis. Total proteome samples were adjusted to a final concentration of 0.2 μg/μl. Samples were analyzed using an EASY‐nLC 1000 (Thermo Fisher) coupled to a Q Exactive, Q Exactive Plus, and an Orbitrap Elite mass spectrometer (Thermo Fisher), respectively. Peptides were separated on 16 cm frit‐less silica emitters (New Objective, 0.75 μm inner diameter), packed in‐house with reversed‐phase ReproSil‐Pur C18 AQ 3 μm resin (Dr. Maisch). Peptides (5 μl) were loaded on the column and eluted for 120 min using a segmented linear gradient of 0% to 95% solvent B (solvent A 5% ACN, 0.5% FA; solvent B 100% ACN, 0.5% FA) at a flow rate of 250 nl/min. Parameters for the different machines are listed in Dataset EV1.

### MS data analysis

Raw data were processed using MaxQuant software version 1.5.2.8 (http://www.maxquant.org/) (Cox & Mann, 2008). MS/MS spectra were searched with the Andromeda search engine against the TAIR10 database (TAIR10_pep_20101214; ftp://ftp.arabidopsis.org/home/tair/Proteins/TAIR10_protein_lists/). Sequences of 248 common contaminant proteins and decoy sequences were automatically added during the search. Trypsin specificity was required, and a maximum of two (proteome) or four missed cleavages (acetylome) were allowed. Minimal peptide length was set to seven amino acids. Carbamidomethylation of cysteine residues was set as fixed, oxidation of methionine, and protein N‐terminal acetylation as variable modifications. Acetylation of lysines was set as variable modification only for the antibody‐enriched samples. Light and medium dimethylation of lysines and peptide N‐termini were set as labels. Peptide–spectrum matches and proteins were retained if they were below a false discovery rate of 1%, modified peptides were filtered for a score ≥ 35 and a delta score of ≥ 6. Match between runs and requantify options were enabled. Downstream data analysis was performed using Perseus version 1.5.5.3 (Tyanova *et al*, 2016). For proteome and acetylome, reverse hits and contaminants were removed, the site ratios log2‐transformed, and flip‐label ratios inverted. For quantitative lysine acetylome analyses, sites were filtered for a localization probability of ≥ 0.75. The “expand site table” feature of Perseus was used to allow separate analysis of site ratios for multiply acetylated peptides occurring in different acetylation states. Technical replicates were averaged, and proteins or sites displaying less than two out of three ratios were removed. The resulting matrices for proteome and acetylome, respectively, were exported and significantly differentially abundant protein groups and lysine acetylation sites were determined using the LIMMA package (Ritchie *et al*, 2015) in R 3.3.1 (R Core Team, 2016). Volcano plots were generated with R base graphics, plotting the non‐adjusted *P*‐values versus the log2 fold‐change and marking data points below 5% FDR (i.e., adjusted *P*‐values, Benjamini–Hochberg) when present.

### Data availability

The raw data, MaxQuant output files, and annotated MS2 spectra for all acetylated peptides have been deposited to the ProteomeXchange Consortium (http://proteomecentral.proteomexchange.org) via the PRIDE partner repository with the dataset identifiers PXD006651, PXD006652, PXD006695, PXD006696.

## Author contributions

MF, MH, IF, AB, MP, KK, MS, PJB, J‐OJ, and JS performed research; JC performed computational analysis; MH, MS, and IF designed research; DS, DL, GU, GBGM, and MM provided reagents and analytical equipment; MF, MH, J‐OJ, KK, and IF analyzed data; IF drafted the manuscript with input from MH, KK, DL, DS, and MES.

## Conflict of interest

The authors declare that they have no conflict of interest.

## Supporting information



Appendix

Dataset EV1

Dataset EV2

Dataset EV3

Dataset EV4

Dataset EV5

Dataset EV6

Review Process File

## References

[msb177819-bib-0001] Alinsug MV , Yu CW , Wu K (2009) Phylogenetic analysis, subcellular localization, and expression patterns of RPD3/HDA1 family histone deacetylases in plants. BMC Plant Biol 9: 37 19327164 10.1186/1471-2229-9-37 PMC2671507

[msb177819-bib-0002] Allfrey VG , Faulkner R , Mirsky AE (1964) Acetylation and methylation of histones and their possible role in regulation of Rna synthesis. Proc Natl Acad Sci USA 51: 786–794 14172992 10.1073/pnas.51.5.786 PMC300163

[msb177819-bib-0003] Ausín I , Alonso‐Blanco C , Jarillo JA , Ruiz‐García L , Martínez‐Zapater JM (2004) Regulation of flowering time by FVE, a retinoblastoma‐associated protein. Nat Genet 3: 162–166 10.1038/ng1295 14745447

[msb177819-bib-0004] Barta C , Carmo‐Silva AE , Salvucci ME (2011) Purification of rubisco activase from leaves or after expression in *Escherichia coli* In Photosynthesis research protocols, Carpentier R (ed.), pp 363–374. Totowa, NJ: Humana Press 10.1007/978-1-60761-925-3_28 20960143

[msb177819-bib-0005] Benhamed M , Bertrand C , Servet C , Zhou DX (2006) *Arabidopsis* GCN5, HD1, and TAF1/HAF2 interact to regulate histone acetylation required for light‐responsive gene expression. Plant Cell 18: 2893–2903 17085686 10.1105/tpc.106.043489 PMC1693931

[msb177819-bib-0006] Boersema PJ , Raijmakers R , Lemeer S , Mohammed S , Heck AJR (2009) Multiplex peptide stable isotope dimethyl labeling for quantitative proteomics. Nat Protoc 4: 484–494 19300442 10.1038/nprot.2009.21

[msb177819-bib-0007] Buchanan BB (2016) The carbon (formerly dark) reactions of photosynthesis. Photosynth Res 128: 215–217 26704182 10.1007/s11120-015-0212-z

[msb177819-bib-0008] Calfapietra C , Penuelas J , Niinemets U (2015) Urban plant physiology: adaptation‐mitigation strategies under permanent stress. Trends Plant Sci 20: 72–75 25476199 10.1016/j.tplants.2014.11.001

[msb177819-bib-0009] Carmo‐Silva AE , Gore MA , Andrade‐Sanchez P , French AN , Hunsaker DJ , Salvucci ME (2012) Decreased CO2 availability and inactivation of Rubisco limit photosynthesis in cotton plants under heat and drought stress in the field. Environ Exp Bot 83: 1–11

[msb177819-bib-0010] Carmo‐Silva AE , Salvucci ME (2013) The regulatory properties of rubisco activase differ among species and affect photosynthetic induction during light transitions. Plant Physiol 161: 1645–1655 23417088 10.1104/pp.112.213348 PMC3613445

[msb177819-bib-0011] Chen LT , Luo M , Wang YY , Wu K (2010) Involvement of *Arabidopsis* histone deacetylase HDA6 in ABA and salt stress response. J Exp Bot 61: 3345–3353 20519338 10.1093/jxb/erq154 PMC2905197

[msb177819-bib-0012] Choi SM , Song HR , Han SK , Han M , Kim CY , Park J , Lee YH , Jeon JS , Noh YS , Noh B (2012) HDA19 is required for the repression of salicylic acid biosynthesis and salicylic acid‐mediated defense responses in *Arabidopsis* . Plant J 71: 135–146 22381007 10.1111/j.1365-313X.2012.04977.x

[msb177819-bib-0013] Choudhary C , Kumar C , Gnad F , Nielsen ML , Rehman M , Walther TC , Olsen JV , Mann M (2009) Lysine acetylation targets protein complexes and co‐regulates major cellular functions. Science 325: 834–840 19608861 10.1126/science.1175371

[msb177819-bib-0014] Cigliano RA , Cremona G , Paparo R , Termolino P , Perrella G , Gutzat R , Consiglio MF , Conicella C (2013) Histone deacetylase AtHDA7 is required for female gametophyte and embryo development in *Arabidopsis* . Plant Physiol 163: 431–440 23878078 10.1104/pp.113.221713 PMC3762662

[msb177819-bib-0015] Clough SJ , Bent AF (1998) Floral dip: a simplified method for Agrobacterium‐mediated transformation of *Arabidopsis thaliana* . Plant J 16: 735–743 10069079 10.1046/j.1365-313x.1998.00343.x

[msb177819-bib-0016] Cox J , Mann M (2008) MaxQuant enables high peptide identification rates, individualized p.p.b.‐range mass accuracies and proteome‐wide protein quantification. Nat Biotechnol 26: 1367–1372 19029910 10.1038/nbt.1511

[msb177819-bib-0017] Dietz KJ (2015) Efficient high light acclimation involves rapid processes at multiple mechanistic levels. J Exp Bot 66: 2401–2414 25573858 10.1093/jxb/eru505

[msb177819-bib-0018] Dose A , Jost JO , Spiess AC , Henklein P , Beyermann M , Schwarzer D (2012) Facile synthesis of colorimetric histone deacetylase substrates. Chem Commun 48: 9525–9527 10.1039/c2cc34422j 22902974

[msb177819-bib-0019] Dose A , Sindlinger J , Bierlmeier J , Bakirbas A , Schulze‐Osthoff K , Einsele‐Scholz S , Hartl M , Essmann F , Finkemeier I , Schwarzer D (2016) Interrogating substrate selectivity and composition of endogenous histone deacetylase complexes with chemical probes. Angew Chem Int Ed Engl 55: 1192–1195 26662792 10.1002/anie.201508174

[msb177819-bib-0020] Drazic A , Myklebust LM , Ree R , Arnesen T (2016) The world of protein acetylation. Bba‐Proteins Proteom 1864: 1372–1401 10.1016/j.bbapap.2016.06.007 27296530

[msb177819-bib-0021] Earley K , Lawrence RJ , Pontes O , Reuther R , Enciso AJ , Silva M , Neves N , Gross M , Viegas W , Pikaard CS (2006) Erasure of histone acetylation by *Arabidopsis* HDA6 mediates large‐scale gene silencing in nucleolar dominance. Gene Dev 20: 1283–1293 16648464 10.1101/gad.1417706 PMC1472903

[msb177819-bib-0022] El Zawily AM , Schwarzlander M , Finkemeier I , Johnston IG , Benamar A , Cao Y , Gissot C , Meyer AJ , Wilson K , Datla R , Macherel D , Jones NS , Logan DC (2014) FRIENDLY regulates mitochondrial distribution, fusion, and quality control in *Arabidopsis* . Plant Physiol 166: 808–828 25165398 10.1104/pp.114.243824 PMC4213110

[msb177819-bib-0023] Fang XP , Chen WY , Zhao Y , Ruan SL , Zhang HM , Yan CQ , Jin L , Cao LL , Zhu J , Ma HS , Cheng ZY (2015) Global analysis of lysine acetylation in strawberry leaves. Front Plant Sci 6: 739 26442052 10.3389/fpls.2015.00739 PMC4569977

[msb177819-bib-0024] Finkemeier I , Laxa M , Miguet L , Howden AJ , Sweetlove LJ (2011) Proteins of diverse function and subcellular location are lysine acetylated in *Arabidopsis* . Plant Physiol 155: 1779–1790 21311031 10.1104/pp.110.171595 PMC3091095

[msb177819-bib-0025] Finkemeier I , König AC , Heard W , Nunes‐Nesi A , Pham PA , Leister D , Fernie AR , Sweetlove LJ (2013) Transcriptomic analysis of the role of carboxylic acids in metabolite signaling in *Arabidopsis* leaves. Plant Physiol 162: 239–253 23487434 10.1104/pp.113.214114 PMC3641205

[msb177819-bib-0026] Gantt SL , Gattis SG , Fierke CA (2006) Catalytic activity and inhibition of human histone deacetylase 8 is dependent on the identity of the active site metal ion. Biochemistry 45: 6170–6178 16681389 10.1021/bi060212u

[msb177819-bib-0027] Gao X , Hong H , Li WC , Yang L , Huang J , Xiao YL , Chen XY , Chen GY (2016) Downregulation of rubisco activity by non‐enzymatic acetylation of RbcL. Mol Plant 9: 1018–1027 27109602 10.1016/j.molp.2016.03.012

[msb177819-bib-0028] Hartl M , Finkemeier I (2012) Plant mitochondrial retrograde signaling: post‐translational modifications enter the stage. Front Plant Sci 3: 253 23162565 10.3389/fpls.2012.00253 PMC3495340

[msb177819-bib-0029] Hartl M , König A‐C , Finkemeier I (2015) Identification of lysine‐acetylated mitochondrial proteins and their acetylation sites In Plant mitochondria: methods and protocols, Whelan J, Murcha MW (eds), pp 107–121. New York, NY: Springer New York 10.1007/978-1-4939-2639-8_7 25910729

[msb177819-bib-0030] He DL , Wang Q , Li M , Damaris RN , Yi XL , Cheng ZY , Yang PF (2016) Global proteome analyses of lysine acetylation and succinylation reveal the widespread involvement of both modification in metabolism in the embryo of germinating rice seed. J Proteome Res 15: 879–890 26767346 10.1021/acs.jproteome.5b00805

[msb177819-bib-0031] Heazlewood JL , Verboom RE , Tonti‐Filippini J , Small I , Millar AH (2007) SUBA: the *Arabidopsis* subcellular database. Nucleic Acids Res 35: D213–D218 17071959 10.1093/nar/gkl863 PMC1635339

[msb177819-bib-0032] Hollender C , Liu ZC (2008) Histone deacetylase genes in *Arabidopsis* development. J Integr Plant Biol 50: 875–885 18713398 10.1111/j.1744-7909.2008.00704.x

[msb177819-bib-0033] Hosp F , Lassowskat I , Santoro V , De Vleesschauwer D , Fliegner D , Redestig H , Mann M , Christian S , Hannah MA , Finkemeier I (2016) Lysine acetylation in mitochondria: from inventory to function. Mitochondrion 33: 58–71 27476757 10.1016/j.mito.2016.07.012

[msb177819-bib-0034] Huber SC (2007) Exploring the role of protein phosphorylation in plants: from signalling to metabolism. Biochem Soc Trans 35: 28–32 17212583 10.1042/BST0350028

[msb177819-bib-0035] Johnova P , Skalak J , Saiz‐Fernandez I , Brzobohaty B (2016) Plant responses to ambient temperature fluctuations and water‐limiting conditions: a proteome‐wide perspective. Bba‐Proteins Proteom 1864: 916–931 10.1016/j.bbapap.2016.02.007 26861773

[msb177819-bib-0036] Joshi P , Greco TM , Guise AJ , Luo Y , Yu F , Nesvizhskii AI , Cristea IM (2013) The functional interactome landscape of the human histone deacetylase family. Mol Syst Biol 9: 672 23752268 10.1038/msb.2013.26 PMC3964310

[msb177819-bib-0037] Jost JO , Hanswillemenke A , Schwarzer D (2015) A miniaturized readout strategy for endogenous histone deacetylase activity. Mol BioSyst 11: 1820–1823 26009008 10.1039/c5mb00326a

[msb177819-bib-0038] Karimi M , Depicker A , Hilson P (2007) Recombinational cloning with plant gateway vectors. Plant Physiol 145: 1144–1154 18056864 10.1104/pp.107.106989 PMC2151728

[msb177819-bib-0039] Kleff S , Andrulis ED , Anderson CW , Sternglanz R (1995) Identification of a gene encoding a yeast histone H4 acetyltransferase. J Biol Chem 270: 24674–24677 7559580 10.1074/jbc.270.42.24674

[msb177819-bib-0040] König AC , Hartl M , Boersema PJ , Mann M , Finkemeier I (2014a) The mitochondrial lysine acetylome of *Arabidopsis* . Mitochondrion 19(Pt B): 252–260. 24727099 10.1016/j.mito.2014.03.004

[msb177819-bib-0041] König AC , Hartl M , Pham PA , Laxa M , Boersema PJ , Orwat A , Kalitventseva I , Plöchinger M , Braun HP , Leister D , Mann M , Wachter A , Fernie AR , Finkemeier I (2014b) The *Arabidopsis* class II sirtuin is a lysine deacetylase and interacts with mitochondrial energy metabolism. Plant Physiol 164: 1401–1414 24424322 10.1104/pp.113.232496 PMC3938629

[msb177819-bib-0042] Kulak NA , Pichler G , Paron I , Nagaraj N , Mann M (2014) Minimal, encapsulated proteomic‐sample processing applied to copy‐number estimation in eukaryotic cells. Nat Methods 11: 319–U300 24487582 10.1038/nmeth.2834

[msb177819-bib-0043] Lassowskat I , Hartl M , Hosp F , Boersema PJ , Mann M , Finkemeier I (2017) Dimethyl‐labeling based quantification of the lysine acetylome and proteome of plants. Methods Mol Biol, photorespiration, AFeaE (ed), 1653: 65–81 28822126 10.1007/978-1-4939-7225-8_5

[msb177819-bib-0044] Lehtimaki N , Koskela MM , Dahlstrom KM , Pakula E , Lintala M , Scholz M , Hippler M , Hanke GT , Rokka A , Battchikova N , Salminen TA , Mulo P (2014) Posttranslational modifications of FERREDOXIN‐NADP(+) OXIDOREDUCTASE in *Arabidopsis* chloroplasts. Plant Physiol 166: 1764–U1917 25301888 10.1104/pp.114.249094 PMC4256869

[msb177819-bib-0045] Maddelein D , Colaert N , Buchanan I , Hulstaert N , Gevaert K , Martens L (2015) The iceLogo web server and SOAP service for determining protein consensus sequences. Nucleic Acids Res 43: W543–W546 25897125 10.1093/nar/gkv385 PMC4489316

[msb177819-bib-0046] Mahrez W , Arellano MST , Moreno‐Romero J , Nakamura M , Shu H , Nanni P , Kohler C , Gruissem W , Hennig L (2016) H3K36ac is an evolutionary conserved plant histone modification that marks active genes. Plant Physiol 170: 1566–1577 26764380 10.1104/pp.15.01744 PMC4775133

[msb177819-bib-0047] Mengel A , Ageeva A , Georgii E , Bernhardt J , Wu K , Durner J , Lindermayr C (2017) Nitric oxide modulates histone acetylation at stress genes by inhibition of histone deacetylases. Plant Physiol 173: 1434–1452 27980017 10.1104/pp.16.01734 PMC5291017

[msb177819-bib-0048] Mignolet‐Spruyt L , Xu EJ , Idanheimo N , Hoeberichts FA , Muhlenbock P , Brosche M , Van Breusegem F , Kangasjarvi J (2016) Spreading the news: subcellular and organellar reactive oxygen species production and signalling. J Exp Bot 67: 3831–3844 26976816 10.1093/jxb/erw080

[msb177819-bib-0049] Nallamilli BRR , Edelmann MJ , Zhong XX , Tan F , Mujahid H , Zhang J , Nanduri B , Peng ZH (2014) Global analysis of lysine acetylation suggests the involvement of protein acetylation in diverse biological processes in rice (*Oryza sativa*). PLoS ONE 9: e89283 24586658 10.1371/journal.pone.0089283 PMC3930695

[msb177819-bib-0050] Newkirk TL , Bowers AA , Williams RM (2009) Discovery, biological activity, synthesis and potential therapeutic utility of naturally occurring histone deacetylase inhibitors. Nat Prod Rep 26: 1293–1320 19779641 10.1039/b817886k

[msb177819-bib-0051] Nunes‐Nesi A , Fernie AR , Stitt M (2010) Metabolic and signaling aspects underpinning the regulation of plant carbon nitrogen interactions. Mol Plant 3: 973–996 20926550 10.1093/mp/ssq049

[msb177819-bib-0052] O'Malley RC , Alonso JM , Kim CJ , Leisse TJ , Ecker JR (2007) An adapter ligation‐mediated PCR method for high‐throughput mapping of T‐DNA inserts in the *Arabidopsis* genome. Nat Protoc 2: 2910–2917 18007627 10.1038/nprot.2007.425

[msb177819-bib-0053] Pandey R , Muller A , Napoli CA , Selinger DA , Pikaard CS , Richards EJ , Bender J , Mount DW , Jorgensen RA (2002) Analysis of histone acetyltransferase and histone deacetylase families of *Arabidopsis thaliana* suggests functional diversification of chromatin modification among multicellular eukaryotes. Nucleic Acids Res 30: 5036–5055 12466527 10.1093/nar/gkf660 PMC137973

[msb177819-bib-0054] Parthun MR , Widom J , Gottschling DE (1996) The major cytoplasmic histone acetyltransferase in yeast: Links to chromatin replication and histone metabolism. Cell 87: 85–94 8858151 10.1016/s0092-8674(00)81325-2

[msb177819-bib-0055] Portis AR Jr , Li C , Wang D , Salvucci ME (2008) Regulation of Rubisco activase and its interaction with Rubisco. J Exp Bot 59: 1597–1604 18048372 10.1093/jxb/erm240

[msb177819-bib-0100] R Core Team (2016) *R: A language and environment for statistical computing* Vienna, Austria: R Foundation for Statistical Computing https://www.R-project.org/

[msb177819-bib-0056] Rappsilber J , Mann M , Ishihama Y (2007) Protocol for micro‐purification, enrichment, pre‐fractionation and storage of peptides for proteomics using StageTips. Nat Protoc 2: 1896–1906 17703201 10.1038/nprot.2007.261

[msb177819-bib-0057] Ritchie ME , Phipson B , Wu D , Hu YF , Law CW , Shi W , Smyth GK (2015) limma powers differential expression analyses for RNA‐sequencing and microarray studies. Nucleic Acids Res 43: e47 25605792 10.1093/nar/gkv007 PMC4402510

[msb177819-bib-0058] Salvucci ME (1992) Subunit Interactions of Rubisco Activase ‐ Polyethylene‐Glycol Promotes Self‐Association, Stimulates ATPase and Activation Activities, and Enhances Interactions with Rubisco. Arch Biochem Biophys 298: 688–696 1416997 10.1016/0003-9861(92)90467-b

[msb177819-bib-0059] Scholz C , Weinert BT , Wagner SA , Beli P , Miyake Y , Qi J , Jensen LJ , Streicher W , McCarthy AR , Westwood NJ , Lain S , Cox J , Matthias P , Mann M , Bradner JE , Choudhary C (2015) Acetylation site specificities of lysine deacetylase inhibitors in human cells. Nat Biotechnol 33: 415–423 25751058 10.1038/nbt.3130

[msb177819-bib-0060] Shen Y , Wei W , Zhou DX (2015) Histone acetylation enzymes coordinate metabolism and gene expression. Trends Plant Sci 20: 614–621 26440431 10.1016/j.tplants.2015.07.005

[msb177819-bib-0061] Smith‐Hammond CL , Hoyos E , Miernyk JA (2014) The pea seedling mitochondrial N‐epsilon‐lysine acetylome. Mitochondrion 19: 154–165 24780491 10.1016/j.mito.2014.04.012

[msb177819-bib-0062] Thimm O , Blasing O , Gibon Y , Nagel A , Meyer S , Kruger P , Selbig J , Muller LA , Rhee SY , Stitt M (2004) MAPMAN: a user‐driven tool to display genomics data sets onto diagrams of metabolic pathways and other biological processes. Plant J 37: 914–939 14996223 10.1111/j.1365-313x.2004.02016.x

[msb177819-bib-0063] Tran HT , Nimick M , Uhrig RG , Templeton G , Morrice N , Gourlay R , DeLong A , Moorhead GBG (2012) *Arabidopsis thaliana* histone deacetylase 14 (HDA14) is an alpha‐tubulin deacetylase that associates with PP2A and enriches in the microtubule fraction with the putative histone acetyltransferase ELP3. Plant J 71: 263–272 22404109 10.1111/j.1365-313X.2012.04984.x

[msb177819-bib-0064] Tyanova S , Temu T , Sinitcyn P , Carlson A , Hein MY , Geiger T , Mann M , Cox J (2016) The Perseus computational platform for comprehensive analysis of (prote)omics data. Nat Methods 13: 731–740 27348712 10.1038/nmeth.3901

[msb177819-bib-0065] Wagner GR , Payne RM (2013) Widespread and enzyme‐independent N‐is an element of‐acetylation and N‐is an element of‐succinylation of proteins in the chemical conditions of the mitochondrial matrix. J Biol Chem 288: 29036–29045 23946487 10.1074/jbc.M113.486753 PMC3790002

[msb177819-bib-0066] Weinert BT , Wagner SA , Horn H , Henriksen P , Liu WSR , Olsen JV , Jensen LJ , Choudhary C (2011) Proteome‐wide mapping of the *Drosophila* acetylome demonstrates a high degree of conservation of lysine acetylation. Sci Signal 4: ra48 21791702 10.1126/scisignal.2001902

[msb177819-bib-0067] Weinert BT , Iesmantavicius V , Wagner SA , Scholz C , Gummesson B , Beli P , Nystrom T , Choudhary C (2013) Acetyl‐phosphate is a critical determinant of lysine acetylation in *E. coli* . Mol Cell 51: 265–272 23830618 10.1016/j.molcel.2013.06.003

[msb177819-bib-0068] Wu FH , Shen SC , Lee LY , Lee SH , Chan MT , Lin CS (2009) Tape‐*Arabidopsis* Sandwich ‐ a simpler *Arabidopsis* protoplast isolation method. Plant Methods 5: 16 19930690 10.1186/1746-4811-5-16 PMC2794253

[msb177819-bib-0069] Wu X , Oh MH , Schwarz EM , Larue CT , Sivaguru M , Imai BS , Yau PM , Ort DR , Huber SC (2011) Lysine acetylation is a widespread protein modification for diverse proteins in *Arabidopsis* . Plant Physiol 155: 1769–1778 21311030 10.1104/pp.110.165852 PMC3091122

[msb177819-bib-0070] Xiong YH , Peng XJ , Cheng ZY , Liu WD , Wang GL (2016) A comprehensive catalog of the lysine‐acetylation targets in rice (Oryza sativa) based on proteomic analyses. J Proteomics 138: 20–29 26836501 10.1016/j.jprot.2016.01.019

[msb177819-bib-0071] Yang XJ , Seto E (2008) Lysine acetylation: codified crosstalk with other posttranslational modifications. Mol Cell 31: 449–461 18722172 10.1016/j.molcel.2008.07.002 PMC2551738

[msb177819-bib-0072] Yuan H , Marmorstein R (2013) Histone acetyltransferases: rising ancient counterparts to protein kinases. Biopolymers 99: 98–111 23175385 10.1002/bip.22128 PMC4017165

[msb177819-bib-0073] Zhang YM , Song LM , Liang WX , Mu P , Wang S , Lin Q (2016) Comprehensive profiling of lysine acetylproteome analysis reveals diverse functions of lysine acetylation in common wheat. Sci Rep 6: 21069 26875666 10.1038/srep21069 PMC4753473

[msb177819-bib-0074] Zhen SM , Deng X , Wang J , Zhu GR , Cao H , Yuan LL , Yan YM (2016) First comprehensive proteome analyses of lysine acetylation and succinylation in seedling leaves of *Brachypodium distachyon* L. Sci Rep 6: 31576 27515067 10.1038/srep31576 PMC4981852

[msb177819-bib-0075] Zheng Y , Ding Y , Sun X , Xie SS , Wang D , Liu XY , Su LF , Wei W , Pan L , Zhou DX (2016) Histone deacetylase HDA9 negatively regulates salt and drought stress responsiveness in *Arabidopsis* . J Exp Bot 67: 1703–1713 26733691 10.1093/jxb/erv562

[msb177819-bib-0076] Zhou CH , Zhang L , Duan J , Miki B , Wu KQ (2005) HISTONE DEACETYLASE19 is involved in jasmonic acid and ethylene signaling of pathogen response in *Arabidopsis* . Plant Cell 17: 1196–1204 15749761 10.1105/tpc.104.028514 PMC1087996

