# Supplementary material for: Lysine acetylome profiling uncovers novel histone deacetylase substrate proteins in Arabidopsis
Source: Mol Syst Biol. 2017 Oct 23;13(10):949. doi: 10.15252/msb.20177819 (PMC5658702; doi:10.15252/msb.20177819)
Supplement: Supplementary file 1 — Appendix [file MSB-13-949-s001.pdf]

APPENDIX

List of Figures

Appendix Figure S1: Multiscatter plots of light (L) and medium (H)-dimethyl-labeled non-normalized protein intensities from inhibitor experiments.

Appendix Figure S2: Venn Diagrams of overlapping quantified acetylation sites and protein groups.

Appendix Figure S3: Up-regulation of lysine acetylation sites (Kac) on Arabidopsis histone tails (H2A, 2B, H3 and H4) upon 4h of 5 µM TSA (blue) and apicidin (red) treatment, respectively.

Appendix Figure S4: Western-blot analysis of HDA14 on isolated chloroplasts and mitochondria.

Appendix Figure S5: Purified recombinant 6xHis-Δ45-HDA14 protein.

Appendix Figure S6: Genotyping and Phenotyping of *hda14* line.

A

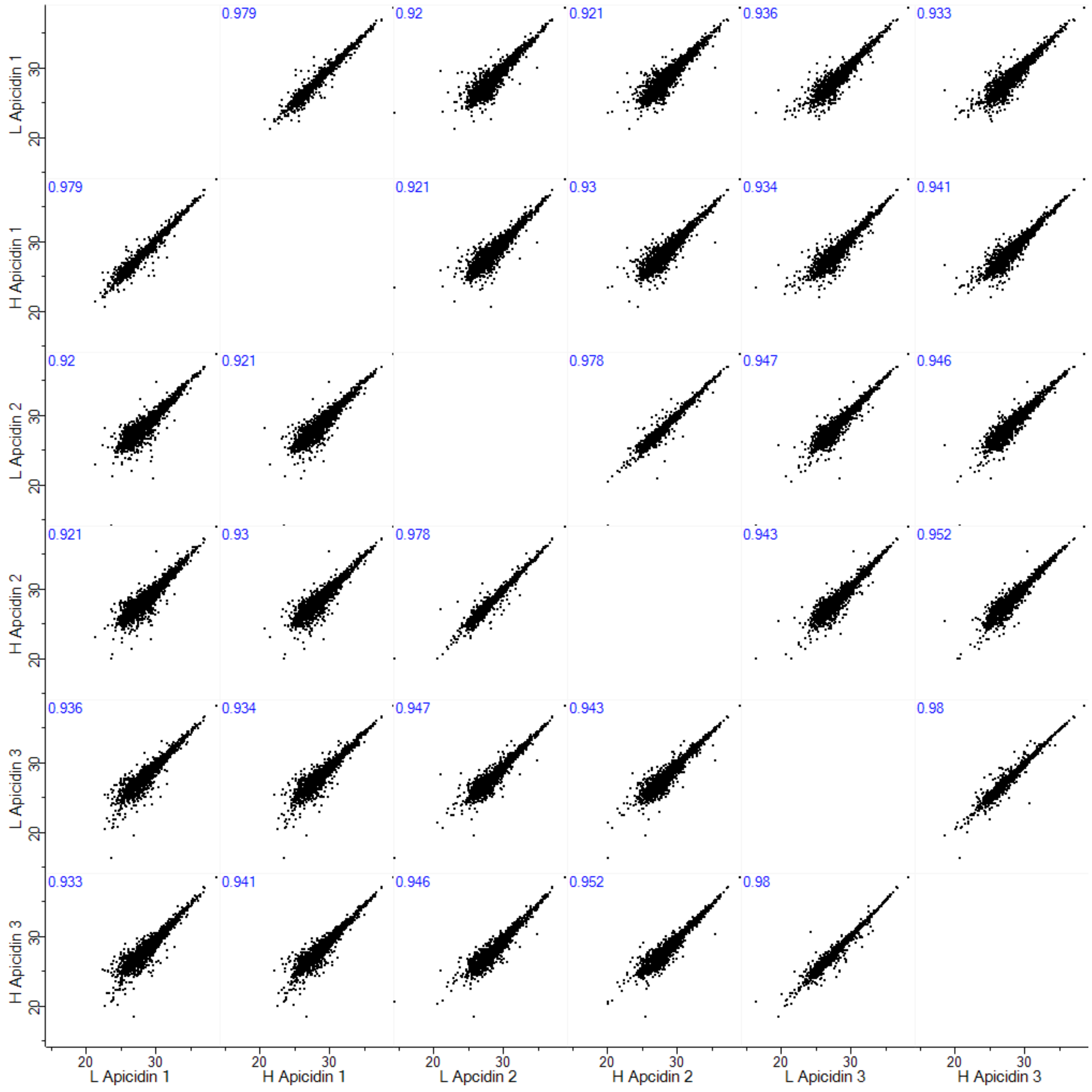

0.979
0.92
0.921
0.936
0.933
0.979
0.921
0.93
0.934
0.941
0.92
0.921
0.978
0.947
0.946
0.921
0.93
0.978
0.943
0.952
0.936
0.934
0.947
0.943
0.98
0.933
0.941
0.946
0.952
0.98
L Apicidin 1
H Apicidin 1
L Apcidin 2
H Apcidin 2
L Apicidin 3
H Apicidin 3
20
30

B

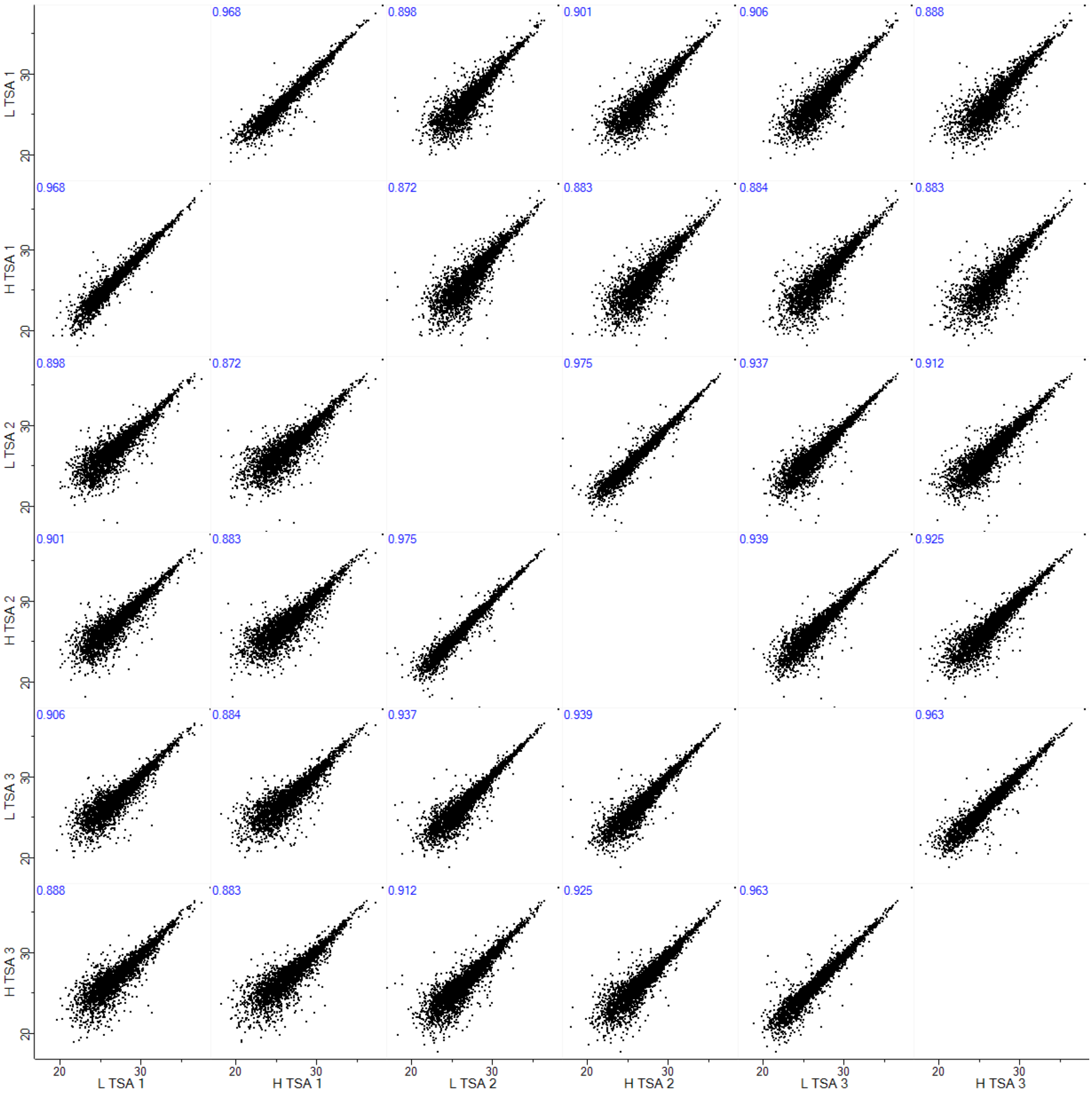

Appendix Figure S1: Multiscatter plots of light (L) and medium (H)-dimethyl-labeled non-normalized protein intensities from inhibitor experiments. (A) apicdin, (B) TSA (n = 3).

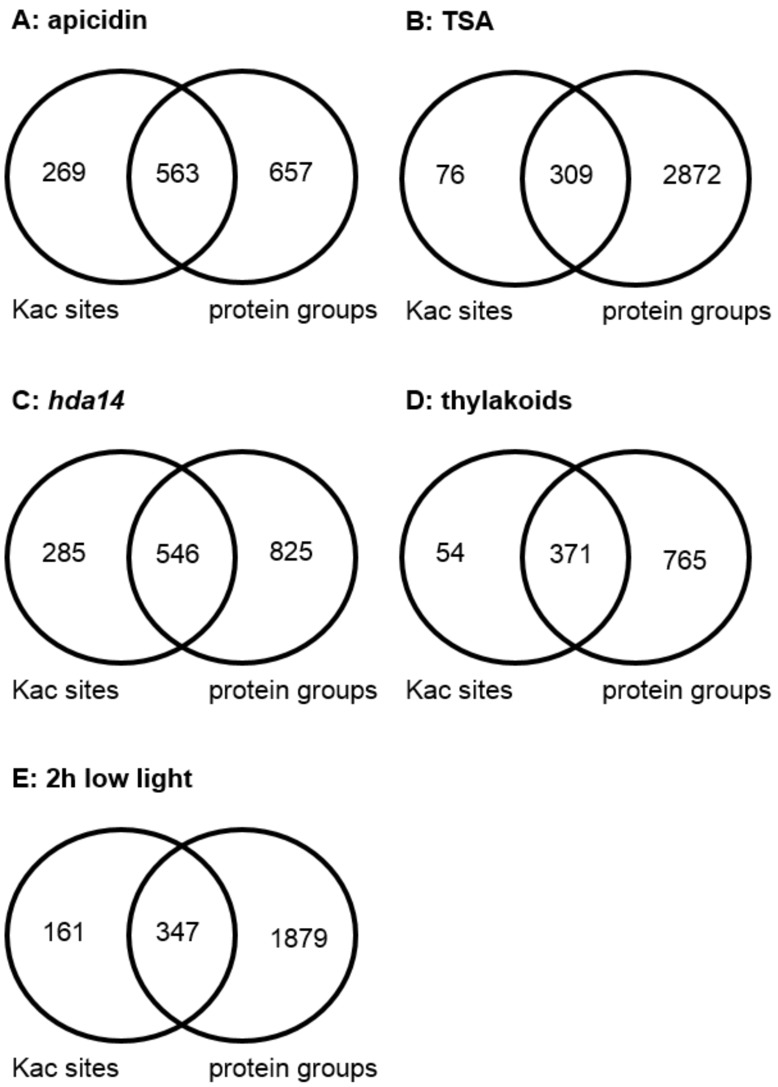

Appendix Figure S2: Venn Diagrams of overlapping quantified acetylation sites and protein groups. Experiments A-E refer to the supplementary datasets EV1-5.

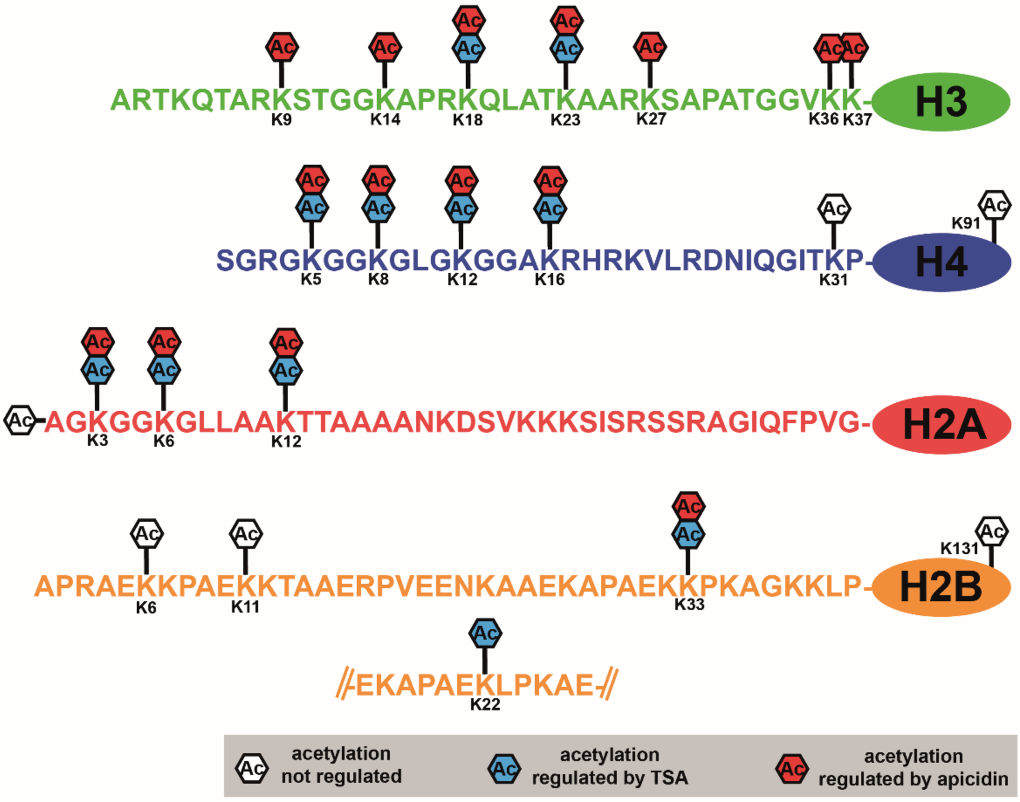

Appendix Figure S3: Up-regulation of lysine acetylation sites (Kac) on Arabidopsis histone tails (H2A, 2B, H3 and H4) upon 4h of 5 µM TSA (blue) and apicidin (red) treatment, respectively. For details on identifications, see Table EV1a and Table EV2a.

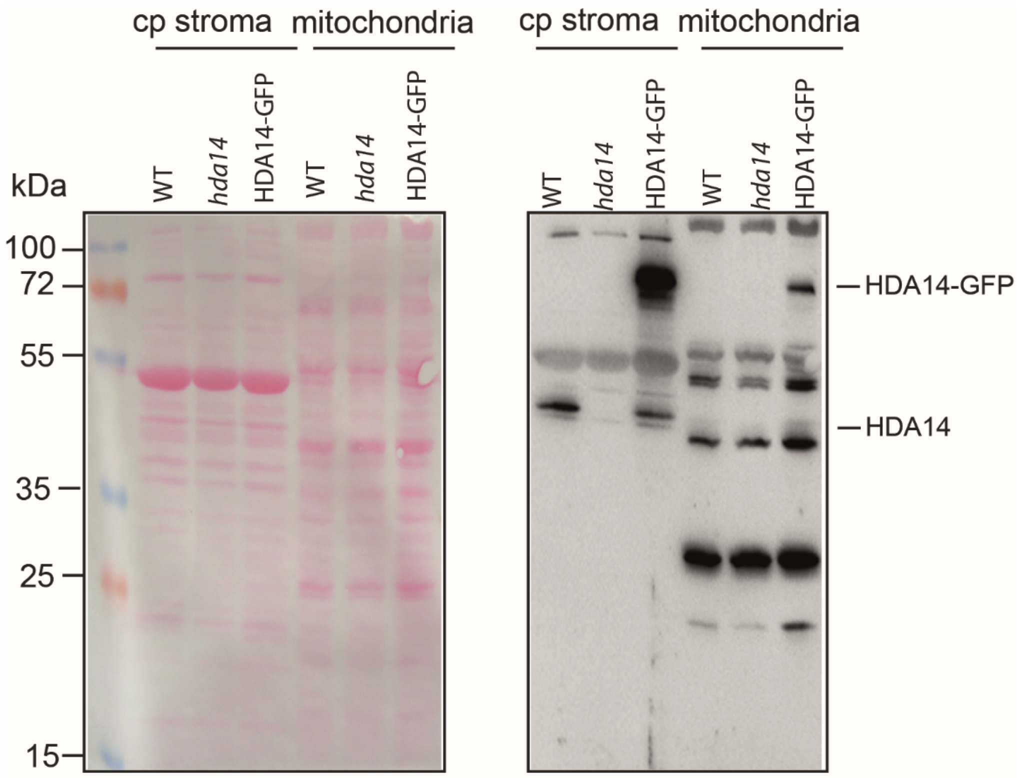

Appendix Figure S4: Western-blot analysis of HDA14 on isolated chloroplasts and mitochondria. Ponceau S stain (left) Western blot analysis (right) of chloroplast (cp) stroma and mitochondria isolated from WT, *hda14* and 35S:HDA14:GFP seedlings. The endogenous HDA14 and HDA14-GFP fusion proteins are indicated. No HDA14 protein was detected in *hda14* seedlings.

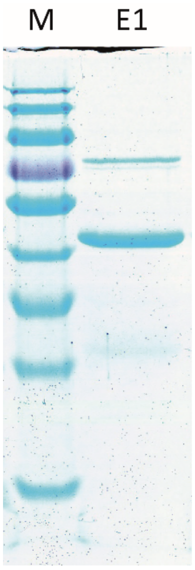

Appendix Figure S5: Purified recombinant 6xHis-Δ45-HDA14 protein. M: Marker, E1: elution fraction 1.

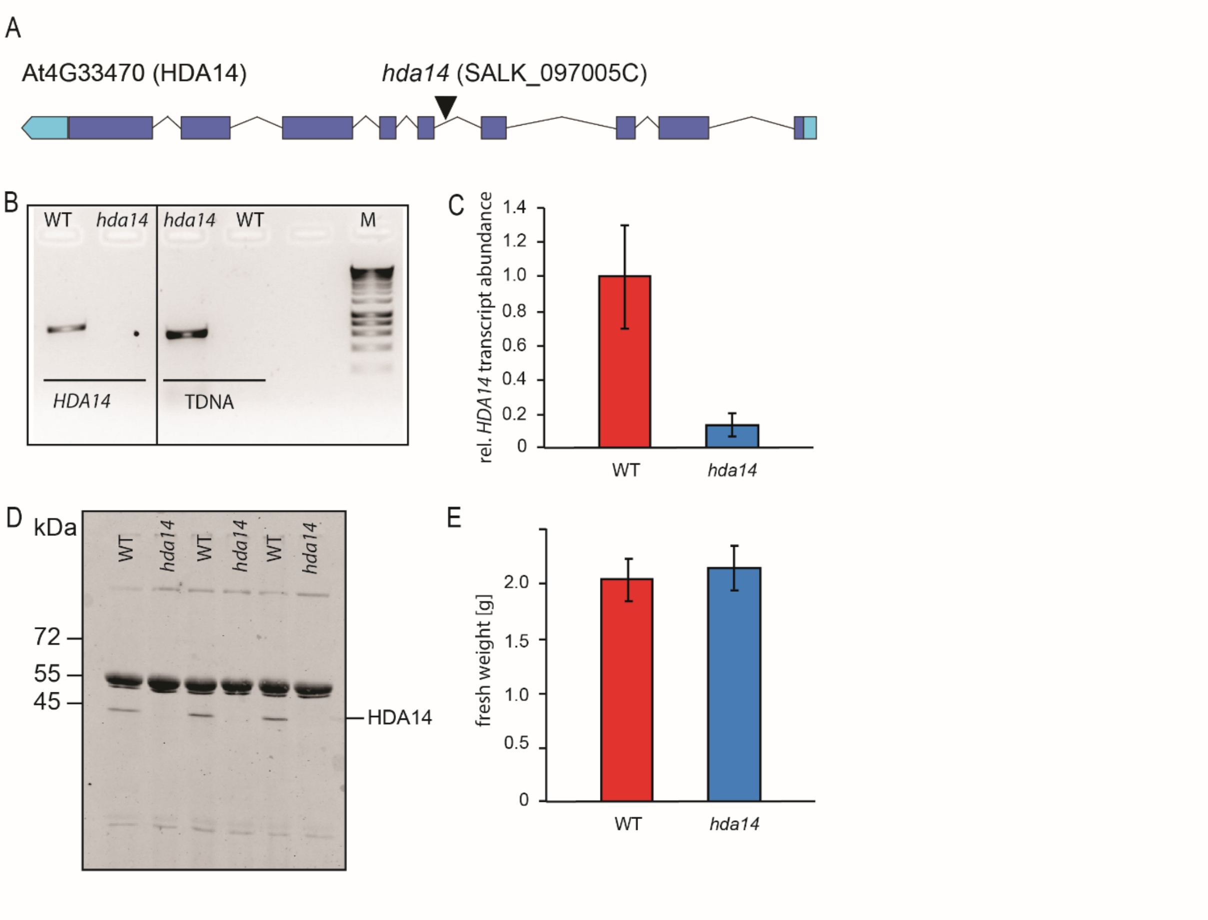

Appendix Figure S6: Genotyping and phenotyping of *hda14* line. (A) Scheme of the HDA14 gene with the TDNA insertion. **(B)** PCR with genomic DNA from WT and *hda14* with primers (HDA14_RP and HDA14_LP) which compose the TDNA insertion site (*HDA14*) and primers flanking the left border of the TDNA (SALK-Lb1.3) and gene specific HDA14_RP primers (TDNA). **(C)** qPCR with cDNA from WT and *hda14* using *HDA14* gene-specific primers. *Actin 2* primers were used as reference, (n = 3, mean ±SD). For primers see materials and methods. **(D)** Western blot analysis with WT and *hda14* protein extracts (n = 3) using an antiserum raised against HDA14 protein (Tran et al,, 2012). **(E)** Fresh weight of 5-week old WT and *hda14* plants (n = 21-27, mean ±SD).
